# Natural variation of root lesion nematode antagonism in the biocontrol fungus *Clonostachys rosea* and identification of biocontrol factors through genome‐wide association mapping

**DOI:** 10.1111/eva.13001

**Published:** 2020-06-02

**Authors:** Mudassir Iqbal, Martin Broberg, Deepak Haarith, Anders Broberg, Kathryn E. Bushley, Mikael Brandström Durling, Maria Viketoft, Dan Funck Jensen, Mukesh Dubey, Magnus Karlsson

**Affiliations:** ^1^ Department of Forest Mycology and Plant Pathology Uppsala BioCenter Swedish University of Agricultural Sciences Uppsala Sweden; ^2^ Department of Plant and Microbial Biology University of Minnesota St. Paul MN USA; ^3^ Department of Molecular Sciences Uppsala BioCenter Swedish University of Agricultural Sciences Uppsala Sweden; ^4^ Department of Ecology Swedish University of Agricultural Sciences Uppsala Sweden

**Keywords:** antagonism, biocontrol, *Clonostachys rosea*, genome‐wide association study, phytopathogenic nematodes, plant growth, wheat

## Abstract

Biological control is a promising approach to reduce plant diseases caused by nematodes to ensure high productivity in agricultural production. Large‐scale analyses of genetic variation in fungal species used for biocontrol can generate knowledge regarding interaction mechanisms that can improve efficacy of biocontrol applications. In this study, we performed a genome‐wide association study (GWAS) for in vitro antagonism against the root lesion nematode *Pratylenchus penetrans* in 53 previously genome re‐sequenced strains of the biocontrol fungus *Clonostachys rosea*. Nematode mortality in *C. rosea* potato dextrose broth (PDB) culture filtrates was highly variable and showed continuous variation (*p* < .001) between strains, indicating a polygenic inheritance. Twenty‐one strains produced culture filtrates with higher (*p* ≤ .05) nematode mortality compared with the PDB control treatment, while ten strains lowered (*p* ≤ .05) the mortality. The difference in in vitro antagonism against *P. penetrans* correlated with antagonism against the soybean cyst nematode *Heterodera glycines*, indicating lack of host specificity in *C. rosea*. An empirical Bayesian multiple hypothesis testing approach identified 279 single nucleotide polymorphism markers significantly (local false sign rate < 10^–10^) associated with the trait. Genes present in the genomic regions associated with nematicidal activity included several membrane transporters, a chitinase and genes encoding proteins predicted to biosynthesize secondary metabolites. Gene deletion strains of the predicted nonribosomal peptide synthetase genes *nps4* and *nps5* were generated and showed increased (*p* ≤ .001) fungal growth and conidiation rates compared to the wild type. Deletion strains also exhibited reduced (*p* < .001) nematicidal activity and reduced (*p* ≤ .05) biocontrol efficacy against nematode root disease and against fusarium foot rot on wheat. In summary, we show that the GWAS approach can be used to identify biocontrol factors in *C. rosea*, specifically the putative nonribosomal peptide synthetases NPS4 and NPS5.

## 1 INTRODUCTION

Nematodes belong to the phylum Nematoda (kingdom Animalia) and comprise > 25,000 known species that display a wide range of feeding habits, including animal‐parasitic, plant‐parasitic, bacterivorous, fungivorous, omnivorous and predatory (Blaxter, 2003). The approximately 4,100 characterized species of plant‐parasitic nematodes are obligate biotrophs and are classified into three major divisions: sedentary endoparasitic, ectoparasitic and semi‐endoparasitic (Decraemer & Hunt, 2013). Plant‐parasitic nematodes are estimated to cause a worldwide average of 14.6% crop losses, equivalent to 157 billion U.S. dollars annually (Abad et al., 2008), but complete loss of marketable yield is not unusual (Barker & Koenning, 1998; Nicol et al., 2011). The most significant economic damage is caused by the sedentary endoparasitic nematodes, including the cyst (*Heterodera* spp. and *Globodera* spp.), root‐knot (*Meloidogyne* spp.) and migratory endoparasitic nematodes (*Pratylenchus* spp.). *Pratylenchus* spp. are also known as root lesion nematodes and attack an extensive variety of crop plants (Bebber, Holmes, & Gurr, 2014; Ganguly & Ganguly, 2008; Moens & Perry, 2009; Williamson & Gleason, 2003), thereby decreasing yield and quality of the crop. Chemical‐based nematicides have been used for controlling plant‐parasitic nematodes. However, this option is now limited due to environmental protection concerns, development of resistance in nematodes (Abad et al., 2008; Viglierchio & Wu, 1989), high costs, toxicity to plants, livestock and biodiversity (Beketov, Kefford, Schäfer, & Liess, 2013). This emphasizes the need for developing effective and environment‐friendly alternatives to chemical nematicides such as biological control.

*Clonostachys rosea* (Link: Fr.) Schroers, Samuels, Seifert & W. Gams, comb. nov. (Schroers, Samuels, Seifert, & Gams, 1999) is a soil‐borne and rhizosphere‐competent fungus, and certain strains can effectively control fungal plant pathogens, including *Alternaria* spp. (Jensen, Knudsen, Madsen, & Jensen, 2004), *Bipolaris sorokiniana* (Jensen, Knudsen, & Jensen, 2002), *Botrytis cinerea* (Sutton et al., 1997), *Fusarium culmorum* (Jensen, Knudsen, & Jensen, 2000), *F. graminearum* (Hue et al., 2009) and *Sclerotinia sclerotiorum* (Rodríguez, Cabrera, Gozzo, Eberlin, & Godeas, 2011). Certain *C. rosea* strains can also antagonize plant‐parasitic nematodes, including *Pratylenchus*, *Heterodera* and *Helicotylenchus* (Iqbal et al., 2019; Iqbal, Dubey, McEwan, et al., 2018; Zou, Tao, et al., 2010; Zou, Tu, Liu, Tao, & Zhang, 2010) and protect carrot and wheat plants from nematode root diseases (Iqbal et al., 2019; Iqbal, Dubey, McEwan, et al., 2018). Several biocontrol mechanisms are described for *C. rosea*, including direct parasitism of fungi (Li, Huang, Kokko, & Acharya, 2002; Yu & Sutton, 1997) and nematodes (Zou, Tao, et al., 2010; Zou, Tu, et al., 2010), antibiosis through the production of secondary metabolites and enzymes (Dong, He, Shen, & Zhang, 2005; Dubey, Jensen, & Karlsson, 2014; Fatema, Broberg, Jensen, Karlsson, & Dubey, 2018; Gan, Yang, Tao, Yu, & Zhang, 2007; Iqbal et al., 2019; Iqbal, Dubey, Gudmundsson, et al., 2018; Iqbal, Dubey, McEwan, et al., 2018; Pachenari & Dix, 1980; Rodríguez et al., 2011; Tzelepis, Dubey, Jensen, & Karlsson, 2015; Zou, Tu, et al., 2010), induction of plant defence reactions (Lahlali & Peng, 2014; Mouekouba et al., 2014; Roberti et al., 2008) and plant growth promotion (Ravnskov et al., 2006; Roberti et al., 2008).

The genome of *C. rosea* strain IK726 was first sequenced using the short‐read Illumina platform technology (Karlsson et al., 2015) and later on supplemented with the long‐read PacBio platform (Broberg et al., 2018). With the advancement of DNA sequencing technology, genome sequencing has become inexpensive and user‐friendly and comparative genomics is now a regular practice in fungal molecular biology (Broberg et al., 2018). Genome‐wide association (GWA) is a powerful technique used to study genetic variants, to identify single nucleotide polymorphisms (SNPs) in different individuals and to pinpoint candidate genes that are associated with a trait of interest in the genome (Bush & Moore, 2012; Korte & Farlow, 2013). GWA studies are reported from the fungal saprotroph *Neurospora crassa* (Palma‐Guerrero et al., 2013) and fungal plant pathogens including *F. graminearum* (Talas, Kalih, Miedaner, & McDonald, 2016), *Heterobasidion annosum* (Dalman et al., 2013), *Parastagonospora nodorum* (Gao et al., 2016) and *Zymoseptoria tritici* (Hartmann, Sánchez‐Vallet, McDonald, & Croll, 2017). In a previous study, we used GWA to identify genomic regions involved in growth rate under cold conditions (10°C) in *C. rosea* (Broberg et al., 2018). However, GWA has not yet been explored to identify genomic regions associated with biological control traits in fungi.

Nonribosomal peptide synthetases (NRPSs) are multipurpose modular enzymes that catalyse the biosynthesis of small nonribosomal peptides by employing the thiotemplate mechanism which is independent of the ribosomal protein synthesis machinery (Finking & Marahiel, 2004; Grünewald & Marahiel, 2006; Reiber et al., 2005; Sieber & Marahiel, 2003). Three functional core domains, that is adenylation or AMP‐binding (A), thiolation (T) and condensation (C) domains, are required to synthesize the peptides (Finking & Marahiel, 2004; Keating, Marshall, Walsh, & Keating, 2002; May, Kessler, Marahiel, & Stubbs, 2002). Many NRPS genes (*nps*) have been identified in sequenced genomes of bacteria and fungi, yet the biological functions of most NRPS remain to be discovered (Bushley & Turgeon, 2010; Oide et al., 2006). To date, only a few functional studies of NRPS in bacteria and fungi have demonstrated their involvement in cell surface properties, growth and reproduction, production of toxins lethal to pathogenic fungi and nematodes, virulence and resistance to oxidative stress (Hahn & Dubnau, 1991; Iqbal et al., 2019; Kim, Cho, La Rota, Cramer, & Lawrence, 2007; Lee et al., 2005; Oide, Krasnoff, Gibson, & Turgeon, 2007; Oide et al., 2006).

In this study, we used a GWA approach to gain insight into the genetic basis of nematode antagonism and biocontrol in *C. rosea*. We found 279 SNP markers associated with in vitro antagonism of the root lesion nematode *P. penetrans*, and genes positioned in the vicinity of these SNPs were predicted to encode several membrane transporters, a chitinase and secondary metabolite biosynthesis proteins (including NRPSs) with possible roles in nematode antagonism. By generating *C. rosea* deletion strains of the *nps4* and *nps5* NRPS genes, we show that NPS4 and NPS5 play a role in growth, conidiation and biocontrol of fusarium foot rot and nematode root disease of wheat.

## 2 MATERIALS AND METHODS

### 2.1 Fungal strains and maintenance conditions

*Clonostachys rosea* strains (Broberg et al., 2018), mutants derived from the *C. rosea* strain IK726 wild type, *B. cinerea* strain B05.10, *F. graminearum* strain PH‐1 and *Rhizoctonia solani* strain SA1 were revived from stock cultures stored in 20% (w/v) glycerol at −80°C and maintained on potato dextrose agar (PDA) medium (Oxoid) at 25°C in darkness.

### 2.2 Antagonism assay using root lesion nematodes

An in vitro assay was performed to determine nematicidal activity in culture filtrates of 53 different *C. rosea* strains. Fifty millilitres potato dextrose broth (PDB; Oxoid) in 300 ml E‐flasks were inoculated with 1 × 10^6^ *C. rosea* conidia/flask and incubated at 25°C on a rotating shaker (120 rpm) under dark conditions. After five days, fungal biomass was separated from the broth by filtering through three layers of filter paper (Ahlstrom‐Munksjö). The culture filtrate was further sterile‐filtered through a 0.45 μm cellulose membrane (Sarstedt), as described previously (Iqbal, Dubey, McEwan, et al., 2018).

*Pratylenchus penetrans* nematodes were purchased from the laboratory of Nematology, Department of Plant Sciences, Wageningen University, Netherlands. Eight hundred microlitres of culture filtrate or fresh liquid media (control) was mixed with 200 μl of water containing approximately 100 *P. penetrans* nematodes in wells of 24‐well plastic plates, followed by incubation at 25°C in darkness for 24 hr. The number of dead nematodes after incubation was determined by washing the nematodes with cold water and by performing the touch assay under a light microscope (Wild M5A Heerbrugg) at 50× magnification (Gill, Olsen, Sampayo, & Lithgow, 2003). The nematode mortality assay was performed on five biological replicates for each *C. rosea* strain.

### 2.3 Test of host specificity of antagonism

Based on the results from the in vitro study with *P. penetrans*, five strains with high antagonism and five with low antagonism were also tested against soybean cyst nematode (SCN; *Heterodera glycines*) to assess *C. rosea* host specificity. In addition to testing for nematicidal activity as with *P. penetrans*, pathogenicity towards SCN eggs, inhibition of egg hatching and juveniles was also investigated.

To assess pathogenicity towards eggs, cysts were extracted from roots of soybean plants grown in the greenhouse, surface‐sterilized with 0.5% NaOCl for three min and placed on two per cent water agar as described previously (Chen, Dickson, & Mitchell, 1996). A five‐mm‐diameter mycelial agar plug of a *C. rosea* strain from a 2‐week‐old PDA culture was transferred to a 9‐cm‐diameter Petri plate containing two per cent water agar and surrounded by seven bacterial‐ and fungal‐free cysts. Three replicates of each fungal strain were compared to three replicates without *C. rosea* inoculation, which served as the control. After twelve days of incubation at 25°C, cysts were removed, and fungal colonization of eggs was determined under the light microscope (phase contrast, Nikon Eclipse C*i*) (Chen & Chen, 2002; Chen et al., 1996). The egg‐parasitic index (EPI) was calculated based on the presence or absence of fungal colonization in individual eggs and summarized on a 0–10 scale as described previously by Chen et al. (1996), in short 0 = no egg colonized, 1 = 1%–10%, 2 = 11%–20%, 3 = 21%–30%, 4 = 31%–40%, 5 = 41%–50%, 6 = 51%–60%, 7 = 61%–70%, 8 = 71%–80%, 9 = 81%–90% and 10 = 91%–100% eggs colonized by mycelia.

To assess inhibition of egg hatching, SCN eggs were collected as described previously (Chen & Chen, 2002; Chen et al., 1996) and surface‐sterilized with an aqueous solution containing 50 ppm streptomycin, 100 ppm chlortetracycline and 50 ppm Fungin. Following 6 hr of incubation at room temperature, the eggs were collected by centrifugation at 2,500 *g* RCF for ten min, rinsed with distilled water twice and stored at 4°C until further use.

Two millilitres *C. rosea* culture filtrates (described above) were supplied into wells of 24‐well plastic plates, while fresh medium and water served as controls. One hundred microlitres of water containing 500 eggs was placed on 1‐cm‐diameter sieves with 35 μm mesh size so that the mesh surface just touched the filtrate solution (Chen et al., 1996). Following incubation at 25°C in darkness for 54 hr, the number of hatched nematodes was determined using an inverted microscope (Nikon Eclipse TS100). Inhibition of egg hatching was calculated as described previously (Chen et al., 1996). Each fungal strain and control treatment was replicated three times.

Stage 2 (J2) juveniles of *H. glycine* were collected by placing eggs on three layers of coffee filter paper (Bunn‐O‐Matic Corporation) and kept on a sterile collecting box. Egg hatching was promoted by moistening the filter paper with 4 mM ZnCl_2_ solution followed by incubation at 27°C for 48 hr. Eight hundred microlitres *C. rosea* culture filtrates (described above) or fresh liquid media (control) was mixed with 200 μl of water containing approximately 100 *H. glycines* J2 juveniles in wells of 24‐well plastic plates, followed by incubation at 25°C in darkness for 24 hr. The number of dead nematodes was determined using an inverted microscope as described above on five biological replicates for each fungal strain.

### 2.4 Genome‐wide association study

Genome‐wide association analysis on *C. rosea* strains was executed using PLINK ver. 1.90 (Purcell et al., 2007), using the parameters—maf 0.1—hwe 1 × 10^–5^ for SNP filtering as described in Broberg et al. (2018). SNPs were annotated using the ANNOVAR software (Wang, Li, & Hakonarson, 2010). Empirical Bayesian multiple hypothesis testing was done with the R package ashr, to estimate local false sign rate (lfsr) (Stephens, 2016). We used a lfsr ≤ 1 × 10^–10^ as a cut‐off for significance.

### 2.5 Sequence and phylogenetic analyses of nonribosomal peptide synthetases

Predicted NRPS proteins were retrieved from the *C. rosea* IK726 genome sequence (Broberg et al., 2018; Karlsson et al., 2015). Identification of conserved protein modules and features was made using the conserved domain database (CDD) (Marchler‐Bauer et al., 2017) and the simple modular architecture research tool (SMART) (Letunic & Bork, 2018). Signal peptides for targeting proteins to the secretory pathway were identified using SignalP ver. 4.1 (Nielsen, 2017).

The A domains of the NRPS proteins were extracted using HMMER ver. 3.1b2 and a custom HMMER model created for fungal A domains (Bushley & Turgeon, 2010). The A domains were aligned with mafft (Katoh, Misawa, Kuma, & Miyata, 2002), along with a reference set of A domains from fungal NRPSs with known chemical products (Bushley & Turgeon, 2010) as outgroups. A maximum likelihood phylogenetic tree was created using RAxML (Stamatakis, Hoover, & Rougemont, 2008) with the best‐fitting protein model (RTREVF) estimated by ProtTest (Abascal, Zardoya, & Posada, 2005). The resulting phylogenetic tree was visualized and annotated using iTOL (https://itol.embl.de/).

### 2.6 Gene expression analysis

For expression analysis of NRPS genes, *C. rosea* was confronted in a dual plate assay with *B. cinerea* (Cr‐Bc), *F. graminearum* (Cr‐Fg) or with itself (Cr‐Cr) as described previously (Dubey, Jensen, & Karlsson, 2016; Iqbal et al., 2019). Seven to ten mm of the growing front of *C. rosea* was harvested at contact with *B. cinerea* or *F. graminearum* mycelia. Mycelium harvested at the same stage in a confrontation of *C. rosea* with itself served as the control treatment. Each treatment was replicated five times.

RNA extraction was carried out using a Qiagen RNeasy kit according to the manufacturer's instruction (Qiagen). RNA was treated with RNase‐free DNase I (Fermentas) and reverse transcribed (RT) in a total volume of 20 μl using the Maxima First‐Strand cDNA Synthesis Kit (Fermentas) followed by 10‐fold dilution and stored at −20°C. Specific PCR primers targeting the *C. rosea* NRPS genes *nps4* and *nps5* (Table S1) were designed using the PrimerSelect software implemented in the DNASTAR Lasergene ver. 10 software package (DNASTAR Inc.). Primer amplification efficiency was determined based on the amplification of serial dilutions of *C. rosea* genomic DNA. Transcript levels were quantified using an iQ5 qPCR system (Bio‐Rad) using the SsoFast EvaGreen Supermix (Bio‐Rad) as described previously (Kamou et al., 2016). A melt curve analysis was performed to confirm that the signal was the result of single product amplification. Relative expression levels were calculated in relation to the reference gene β‐tubulin (*tub*) (Mamarabadi, Jensen, Jensen, & Lübeck, 2008) using the 2^–ΔΔ^ *^C^* ^T^ method (Livak & Schmittgen, 2001) with five biological replicates, each based on two technical replicates.

### 2.7 Construction of gene deletion cassette, transformation and mutant validation deletion

To study the function of *nps4* and *nps5*, deletion strains were constructed. A three‐fragment multisite gateway cloning system (Invitrogen) was used to construct *nps4* and *nps5* gene deletion cassettes. PCR amplification of ~2 kb of the 5′ flank and the 3′ flank regions of both *nps4* and *nps5* genes was performed using *C. rosea* genomic DNA and the gene‐specific primer pairs NPS4‐ups F/ups R, NPS4‐ds F/ds‐R, NPS5‐ups F/ups R and NPS5‐ds F/ds‐R (Table S1), respectively, as shown in Figures S1 and S2. Gateway entry clones of the purified 5′ flank and 3′ flank PCR fragments were generated according to the manufacturer's instruction (Invitrogen). We used a gateway entry clone for the hygromycin‐resistance cassette (hygB) that was constructed during our previous studies (Dubey, Broberg, Jensen, & Karlsson, 2013; Dubey, Broberg, Sooriyaarachchi, et al., 2013; Dubey, Ubhayasekera, Sandgren, Jensen, & Karlsson, 2012). The gateway LR recombination reaction was carried out using the entry plasmid of the respective fragments and the destination vector pPm43GW (Karimi, De Meyer, & Hilson, 2005) to generate a deletion cassette following the conditions as described by the manufacturer (Invitrogen).

*Clonostachys rosea* IK726 was transformed using *Agrobacterium tumefaciens*‐mediated transformation (ATMT) as described previously (Utermark & Karlovsky, 2008). Putative transformants were selected and sub‐cultured on PDA plates containing hygromycin (200 μg/ml) and were validated using primer combinations specific to the hygB cassette (Hyg F/Hyg R) and sequences flanking the deletion vector (NPS4‐ko F and NPS4‐ko R, NPS5‐ko F and NPS5‐ko R) (Table S1) as described previously (Dubey et al., 2014, 2016; Iqbal et al., 2019). RT‐PCR analysis was performed on *C. rosea* wild‐type, *nps4* and *nps5* deletion strains using a *iScript*™ cDNA synthesis kit (Bio‐Rad) and primers specific for *nps4* and *nps5* (NPS4 F_val and NPS4 R_val; NPS5 F_val and NPS5 R_val), the hygromycin‐resistance gene *hph* (HygF_qPCR and HygR_qPCR) and actin (*act*, ActinF_qPCR and ActinR_qPCR) (Table S1). PCR positive transformants were tested for mitotic stability and then purified by single spore isolation as described previously (Dubey et al., 2014, 2016). The length of the *nps4* and *nps5* genes prevented the construction of gene complementation cassettes, and therefore, all phenotypic analyses were performed with five independent positive transformants to ensure that any phenotypic effect was due to the *nps4* and *nps5* gene deletion and not due to ectopic insertions.

### 2.8 Phenotypic analyses of *C. rosea nps4* and *nps5* deletion strains

Growth rate and conidiation of *C. rosea* wild‐type, Δ*nps4* and Δ*nps5* strains were determined following procedure described previously (Fatema et al., 2018; Iqbal et al., 2019).

A dual plate confrontation assay was performed to determine the in vitro antagonistic ability of *C. rosea* strains against *B. cinerea*, *F. graminearum* and *R. solani* (Dubey et al., 2016; Iqbal et al., 2019). In addition, a culture filtrate test was used to measure the in vitro antagonistic ability against *B. cinerea*, *F. graminearum*, *R. solani* and *P. penetrans* nematodes. Culture filtrates were prepared by inoculating 100 ml of PDB in 500 ml E‐flasks with 2 × 10^6^ *C. rosea* conidia/flask, followed by incubation at 25°C in darkness on a rotating shaker (120 rpm) for 2 weeks (Dubey et al., 2014; Fatema et al., 2018; Iqbal et al., 2019). Antagonistic ability against other fungi was determined by measuring their mycelial biomass, while nematicidal activity was determined by counting the number of dead nematodes as described previously (Gill et al., 2003; Iqbal et al., 2019).

To examine the involvement of NPS4 and NPS5 in secondary metabolite production, the culture filtrates mentioned above were further analysed by ultra‐high performance liquid chromatography–mass spectrometry (UHPLC‐MS). UHPLC‐MS analysis was performed using an Agilent 1290 Infinity II UHPLC (Agilent) hyphenated with a Bruker maXis Impact QTOF MS (Bruker Daltonic GmbH.). Samples (1 μl) were analysed on a reversed‐phase UHPLC column (2.1 × 50 mm, 1.5 μm, Accucore Vanquish, Thermo Scientific), eluted with a gradient of acetonitrile (MeCN) in water, both with 0.2% formic acid (10%–95% MeCN in 3 min and 95% MeCN for 1.2 min at 0.9 ml/min). The MS was used in a positive mode in the range m*/z* 50–1,500. Compass Data Analysis 4.3 software (Bruker Daltonics) was used to calibrate mass spectra against sodium formate clusters, to create extracted‐ion chromatograms corresponding to compounds known to be produced by *Clonostachys* and to convert data to mzXML format. Ion‐chromatogram peak picking was conducted using the web‐based platform XCMS Online (Tautenhahn, Boettcher, & Neumann, 2008). The in vitro antagonism assays and chemical analyses were performed on five biological replicates while mycelial biomass production measurements were carried out with three biological replicates.

### 2.9 Assay for biological control of fusarium foot rot disease

An assay for biological control of fusarium foot rot on wheat (*Triticum aestivum*, winter wheat variety “Stava”) was carried out using five biological replicates. Each replicate consisted of 12–15 plants, following a procedure described previously (Dubey et al., 2014, 2016; Knudsen, Hockenhull, & Jensen, 1995). In short, surface‐sterilized wheat seeds were coated with *C. rosea* wild‐type, Δ*nps4* or Δ*nps5* deletion strain conidia (1 × 10^7^ conidia/ml) and were sown in moistened sand in individual plastic pots (5 × 5 × 5 cm). Water‐soaked seeds were used as a control. The pathogen was inoculated by placing a 5‐mm‐diameter PDA plug with *F. graminearum* mycelium in a hole close to the seeds. A sterile PDA plug without any *F. graminearum* mycelium was used as a control. After inoculation, holes were covered with moist sand. Pots were maintained in a growth cabinet under controlled conditions: that is light/dark photoperiod 12 hr/12 hr with 150 μmol/m^2^ s^−1^ light intensity, a temperature of 15°C ± 1°C and a relative humidity of 80 ± 5%. Seedlings were harvested three weeks postinoculation, and disease symptoms were scored based on a 0–4 scale as described previously (Dubey et al., 2014, 2016; Knudsen et al., 1995).

### 2.10 Assay for biological control of nematode root disease

An assay of nematode root disease in wheat was performed with eight biological replicates for each *C. rosea* strain, with the exception for strains 1,830 and CBS 188.33 where only three biological replicates were included. Naturally, nematode‐infested soil containing a diverse community of nematodes was collected from an agricultural field (coordinates: 59°51'10.98"N, 17°40'37.344"E) close to Uppsala, Sweden. Soil was collected from five different locations during fallow conditions and prepared for experiment as described previously (Iqbal et al., 2019; Iqbal, Dubey, McEwan, et al., 2018). Formulations of *C. rosea* strains (five with high in vitro antagonism and five with low in vitro antagonism against *P. penetrans*) and *C. rosea* IK726 wild‐type, Δ*nps4* and Δ*nps5* strains were prepared. Inoculum concentration was determined by counting colony‐forming units (CFUs) and mixing inoculum with soil to reach a final concentration of 2.5 × 10^5^ CFU/g of soil (Iqbal, Dubey, McEwan, et al., 2018; Jensen et al., 2000). Plastic pots (9 × 9 × 10 cm) were filled with 500 g of *C. rosea*‐inoculated or uninoculated soil (control). Eight wheat seeds (*cv*. Stava) were planted in each pot (replicate) and placed in a growth cabinet under controlled conditions as described previously (Iqbal et al., 2019; Iqbal, Dubey, McEwan, et al., 2018). All pots were subjected to an equal amount of water throughout the 7‐week experimental period. At the end of the experiment, plants were cut at the root‐shoot junction at the surface of the soil, and plant dry shoot weight (g) and shoot length (cm) were recorded. Living nematodes were extracted from fifteen g of soil and one g of harvested roots using a modified Baermann funnel method (Baermann, 1917; Viketoft, Palmborg, Sohlenius, Huss‐Danell, & Bengtsson, 2005). Nematodes were counted using a light microscope (Wild M5A Heerbrugg) and were classified to the genus level based on an analysis of morphological characters.

### 2.11 Statistical analyses

Gene expression and phenotypic data were analysed by analysis of variance (ANOVA) using a general linear model approach implemented in Minitab® Statistical Software ver. 18.1 (Minitab Inc.). Pairwise comparisons were performed using Fisher's least significant difference or the Tukey–Kramer method at the 95% significance level. Statistical analysis of UHPLC‐MS data was performed using MetaboAnalyst 4.0, a web‐based tool suite for metabolomic data analysis (Chong et al., 2018). Following sample normalization by sum and log transformation, differences between sample groups (wild type and mutants) were analysed by principal component analysis (PCA) and *t* tests.

## 3 RESULTS

### 3.1 Screening of *C. rosea* strains for in vitro antagonism against nematodes

**FIGURE 1:**
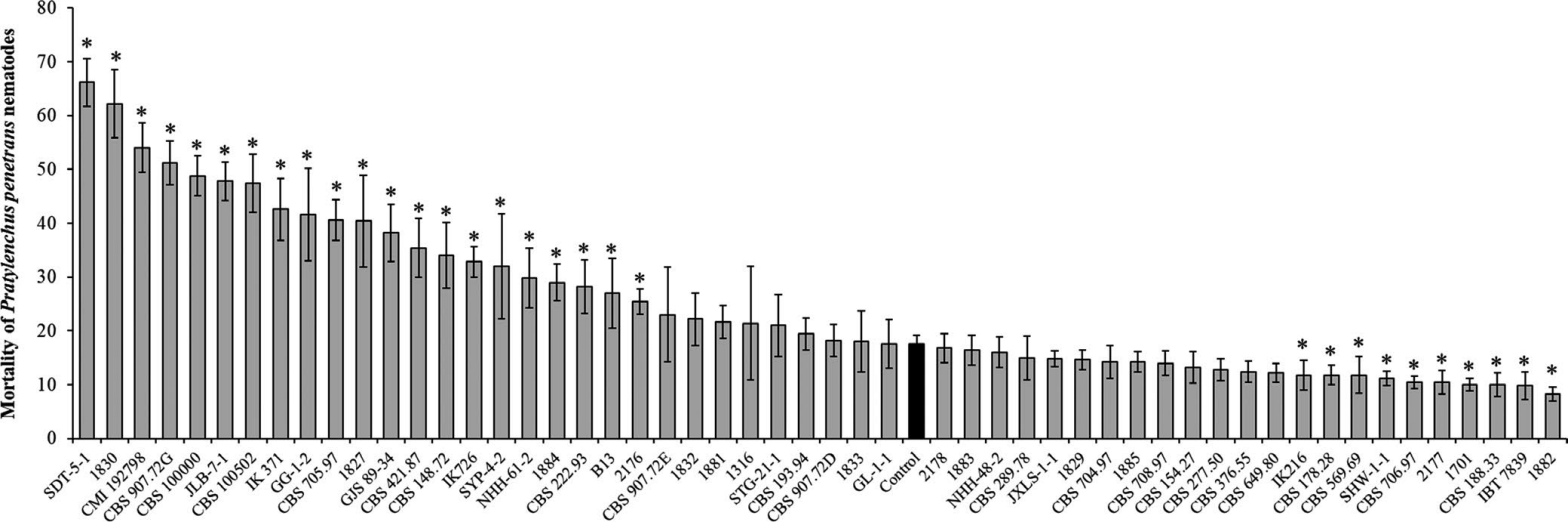
Mortality of *Pratylenchus penetrans* nematodes in potato dextrose broth culture filtrates from 53 *Clonostachys rosea* strains. Nematode mortality was assessed in culture filtrates from *C. rosea* strains and in a control medium (black bar) after 24 hr of incubation at 25°C. Error bars represent the standard deviation based on five biological replicates. Asterisks indicate a significant difference (*p* ≤ .05) between a *C. rosea* culture filtrate and the potato dextrose broth control treatment, as determined by Fisher's least significant difference

After 24 hr of incubation, there were significant (*p* < .001) differences in *P. penetrans* nematode mortality in culture filtrates from the different *C. rosea* strains (Figure 1). Culture filtrates from 21 strains resulted in a significantly (*p* ≤ .05) higher nematode mortality (25%–66%) compared with the PDB medium control where 18% of the nematodes were deceased (Figure 1). Culture filtrates from ten strains showed significantly (*p* ≤ .05) lower nematode mortality levels (8%–12%) compared with the PDB control. Nematode mortality in culture filtrates from the remaining 23 strains was not significantly different from the PDB control (Figure 1).

### 3.2 Host specificity of *C. rosea* in vitro antagonism

**FIGURE 2:**
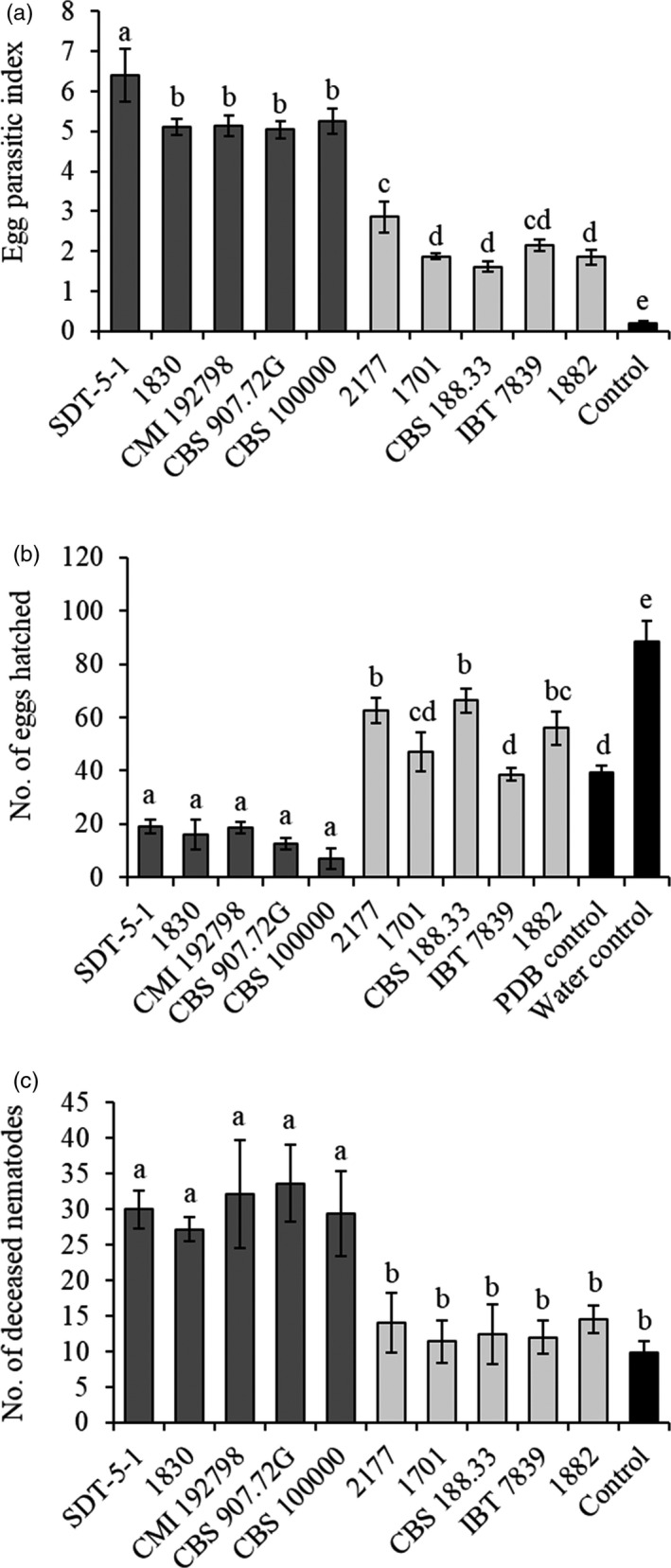
Effect of *Clonostachys rosea* strains on soybean cyst nematode, including egg parasitism, egg hatch inhibition and juvenile mortality. (a) Egg‐parasitic index (EPI) was determined on water agar plates and calculated based on a previously established scale (Chen et al., 1996). (b) The number of soybean cyst nematode eggs hatched in *C. rosea* potato dextrose broth (PDB) culture filtrates. Egg hatching inhibition was assessed after 54 hr of incubation at 25°C. (c) Soybean cyst nematode juveniles (J2) mortality in *C. rosea* PDB culture filtrates. Juvenile mortality was assessed after 24 hr of incubation at 25°C. Error bars represent the standard deviation based on three biological replicates in the case of soybean cyst nematode EPI (a) and eggs hatched (b) and five biological replicates in case of juvenile mortality (c). Different letters indicate a statistically significant difference (*p* ≤ .05) between treatments within an experiment as determined by the Tukey–Kramer test. Dark and light grey columns correspond to high and low antagonism strains, respectively, while black column corresponds to control treatment

Five *C. rosea* strains (SDT‐5‐1, 1830, CMI192798, CBS 907.72G and CBS 100000) with high in vitro antagonism against *P. penetrans* (49%–66% mortality) and five strains (2177, 1701, CBS 188.33, IBT7839 and 1882) with low in vitro antagonism (8%–10% mortality) were evaluated for their ability to antagonize the soybean cyst nematode, *H. glycines*. All strains were able to colonize *H. glycine* eggs, indicated by a significantly (*p* ≤ .05) higher EPI compared with the control treatment (Figure 2a). Strains with a high in vitro antagonism against *P. penetrans* also displayed a higher (*p* < .001) level of parasitism of SCN eggs (EPI = 5.5 ± 0.6 [standard deviation]) compared with strains with a low in vitro antagonism against *P. penetrans* (EPI = 2.1 ± 0.5).

Culture filtrates of strains with a high in vitro antagonism against *P. penetrans* also inhibited hatching of SCN eggs (*p* ≤ .05) compared with the PDB control treatment (Figure 2b). In contrast, strains with a low in vitro antagonism against *P. penetrans* were not able to inhibit egg hatching. In fact, strains CBS 188.33, 2177 and 1882 increased (*p* ≤ .05) hatch rate by 42%–69% compared with the PDB control (Figure 2b). Egg hatching in the PDB control was 55% lower (*p* ≤ .05) compared with egg hatching in water only.

Culture filtrates of *C. rosea* strains with a high in vitro antagonism against *P. penetrans* also resulted in a higher (*p* ≤ .05) mortality of SCN juveniles, compared with the PDB control treatment (Figure 2c). This was not the case in culture filtrates from strains with a low in vitro antagonism against *P. penetrans*, where no differences in mortality compared with the control were detected.

### 3.3 Correlation between in vitro antagonism and biocontrol of nematode root disease

**FIGURE 3:**
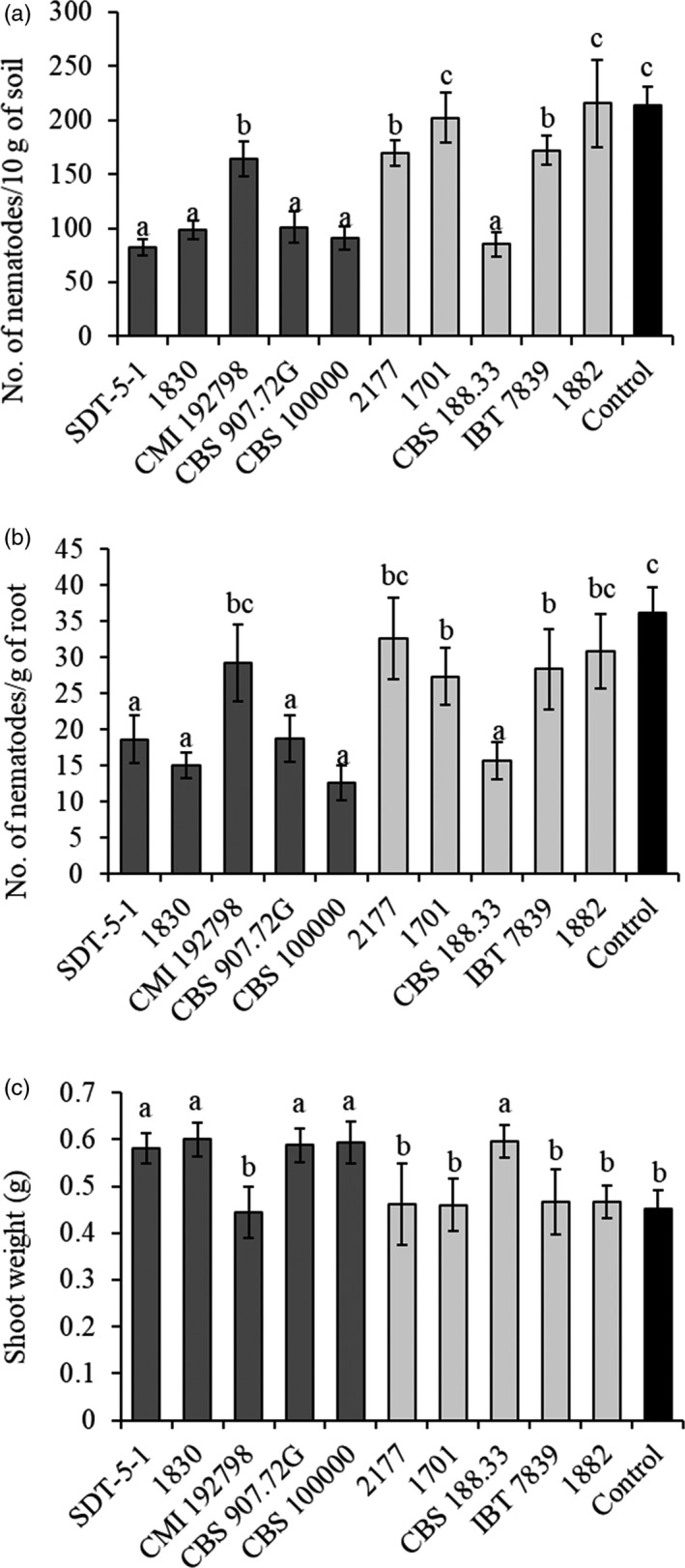
Effect of soil application of *Clonostachys rosea* strains on nematode populations and shoot weight of wheat. (a) Effect of *C. rosea* strains on nematode populations recovered from soil including plant‐parasitic nematode genera (*Pratylenchus*, *Helicotylenchus*, *Tylenchorhynchus*, *Merlinus*, *Boleodorus*, *Heterodera* (J2) and *Paratylenchus*) and other trophic groups (bacterivorous, fungivorous and omnivorous). (b) Effect of *C. rosea* strains on nematode populations recovered from wheat roots. The data include both migratory endoparasitic nematodes from the genus *Pratylenchus* and endoparasitic nematodes from the genus *Heterodera* (J2). The control treatment corresponds to plants grown in naturally nematode‐infested soil without *C. rosea* application. (c) Effect of soil application of *C. rosea* on dry shoot weight (g) of plants grown in soil treated with *C. rosea* or in soil that was not treated with a *C. rosea* application (control). Error bars represent the standard deviation based on eight biological replicates, with the exception for strains 1,830 and CBS 188.33 where only three biological replicates were scored. Different letters indicate a statistically significant difference (*p* ≤ .05) between treatments as determined by Fisher's least significant difference in (a) and by the Tukey–Kramer test in (b) and (c). Dark and light grey columns correspond to high and low antagonism strains, respectively, while black column corresponds to control treatment

The five *C. rosea* strains with high in vitro antagonism against *P. penetrans* and the five strains with low in vitro antagonism indicated above were further evaluated for their ability to control nematode root disease on wheat using a pot experiment. All *C. rosea* strains, except 1701 and 1882, were able to reduce (*p* ≤ .05) the number of nematodes belonging to plant‐parasitic genera (*Pratylenchus*, *Helicotylenchus*, *Tylenchorhynchus*, *Merlinus*, *Boleodorus*, *Heterodera* and *Paratylenchus*) recovered from soil (Figure 3a). The average number of plant‐parasitic nematodes recovered from pots inoculated with strains showing high in vitro antagonism was 107 ± 33 compared with 169 ± 51 from pots inoculated with strains showing low in vitro antagonism. This difference was significant at *p* = .052.

All *C. rosea* strains, except CMI192798, 2177 and 1882, were effective in reducing (*p* ≤ .05) *Pratylenchus* and *Heterodera* nematodes recovered from wheat roots (Figure 3b). The difference between strains with high and low in vitro antagonistic ability (19 ± 6 versus 27 ± 7 nematodes per pot) was not significant (*p* = .084).

Soil inoculation with *C. rosea* strains SDT‐5‐1, 1830, CBS 907.72G, CBS 100000 and CBS 188.33 increased (*p* ≤ .05) dry shoot weight of the wheat plants by on average 31%, compared with the control treatment (Figure 3c). However, dry shoot weight of wheat plants did not differ (*p* = .110) between *C. rosea* strains with high and low in vitro antagonistic ability.

### 3.4 Identification of *C. rosea* SNPs associated with in vitro antagonism to nematodes

The GWA analysis of the 53 *C. rosea* strains and empirical Bayesian multiple hypothesis correction identified a total of 279 SNPs that were associated with in vitro antagonism against *P. penetrans* at lfsr ≤ 1 × 10^–10^. Out of these, 193 SNPs were located in intergenic regions, 29 in exonic regions, five in intronic regions and seven in the 5′ or 3′ untranslated regions (Table S2).

Several SNPs were identified close (exonic, intronic or within 600 bp up‐ or downstream) to genes putatively encoding major facilitator superfamily (MFS) transporters CRV2G00021063, CRV2G00021188 and CRV2G00021796, being members of MFS transporter families 2.A.1.2.33 (unknown function), 2.A.1.14.11 (nicotinate permease) and 2.A.1.2.85 (peroxisomal phenylacetate transporter), respectively. One SNP was located 549 bp downstream of CRV2G00020959, predicted to encode the ATP‐binding cassette (ABC) transporter ABCB14 (Karlsson et al., 2015). One SNP was located 21 bp upstream of CRV2G00012293, predicted to encode the CHIA4 chitinase (Tzelepis et al., 2015). SNPs unitig_1:417,862 and unitig_306:5,647 were associated with the predicted fungal‐type transcription factors CRV2G00013596 and CRV2G00021935, respectively. Three SNPs were associated with a predicted TfdA‐family oxidoreductase (CRV2G00021724), a predicted decarboxylase (CRV2G00022270) and a predicted cytochrome P450 (CRV2G00019702), respectively, all implicated in biosynthesis of secondary metabolites in other fungal species. SNP unitig_347:5,746 was located in an exon of CRV2G00021286, predicted to encode a NRPS. By relaxing the lfsr cut‐off for significance, two additional SNPs associated with predicted NRPSs were identified, unitig_346:7,460 located 1,009 bp from CRV2G00021335 (lfsr = 0.002) and the intronic unitig_246:8,741 in CRV2G00021642 (lfsr = 0.017; Table S2).

### 3.5 Sequence analyses of NRPS genes

Comparisons between the *C. rosea* IK726 genome ver. 1 (Karlsson et al., 2015) and ver. 2 (Broberg et al., 2018) revealed that CRV2G00021286 and CRV2G00021335 represented partial fragments of the predicted NPS5 NRPS (Karlsson et al., 2015). CRV2G00021642 represented a partial fragment of the predicted NPS4 NRPS (Karlsson et al., 2015). The open reading frames of *nps4* and *nps5* were 38,358 and 16,800 bp, respectively, and were predicted to encode polypeptides composed of 8,453 (protein ID CRV2T00004776) and 5,443 (protein ID CRV2T00009763) amino acid residues, respectively. Conserved domain and SMART analyses of the translated amino acid sequences showed that both NPS4 and NPS5 included three domains necessary for their NRPS function, that is A, T and C domains. SignalP analysis did not identify an N‐terminal secretion signal peptide in either NPS4 or NPS5. NPS4 contained eleven complete modules of A‐T‐C domains, while NPS5 contained five.

**FIGURE 4:**
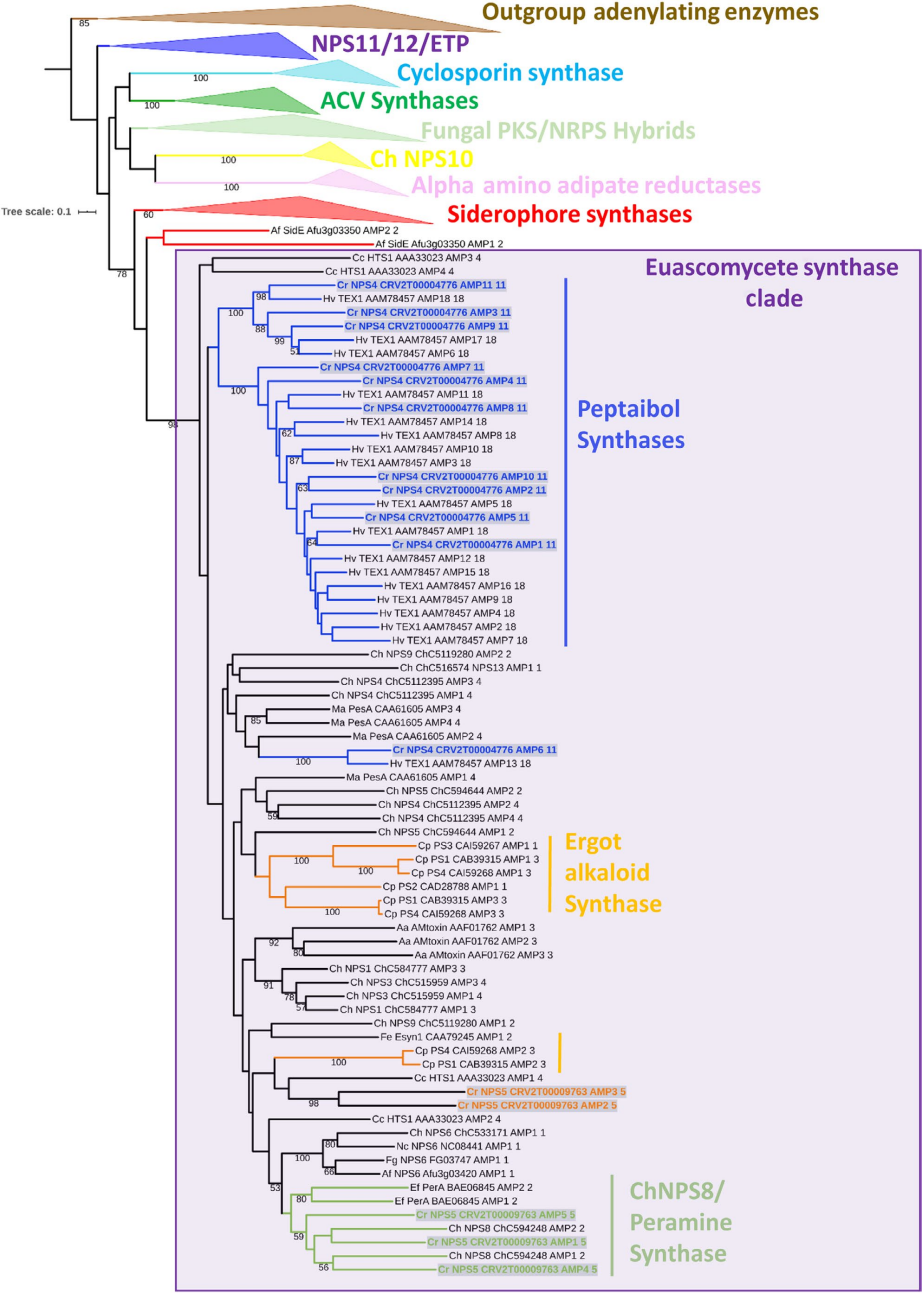
Maximum likelihood phylogenetic tree including *Clonostachys rosea* NPS4 and NPS5 within the context of previously identified clades of NRPSs in fungi (Bushley & Turgeon, 2010). Major groups are shown as collapsed clade including outgroup adenylating enzymes (brown) NPS11/12/ETP (dark blue), cyclosporin synthases (turquoise), ACV synthases (dark green), fungal PKS‐NRPS hybrids (light green), ChNPS10 (yellow), alpha‐amino adipates (pink), siderophore synthases (red) and the Euascomycete clade (purple box). Both Cr NPS4 and Cr NPS5 fell within the euascomycete clade (purple box), which contained primarily multimodular NRPSs that are expanded in euascomycete fungi. Within the euascomycete clade, the eleven A domains of NPS4 grouped within peptaibol synthases (dark blue branches with *C. rosea* NPS4 A domains highlighted), suggesting this NRPS may produce an eleven amino acid peptaibol. Three of the *C. rosea* NPS5 A domains grouped within the ChNPS8/peramine synthase clade (light green with *C. rosea* NPS5 A domains highlighted). The remaining two A domains of this gene grouped adjacent to the second A domain of PS1 and PS4, two genes involved in the biosynthesis of ergot alkaloids (orange with *C. rosea* NPS5 A domain highlighted)

Phylogenetic analysis of the two *C. rosea* NRPSs with other known fungal NRPSs showed that both of these NRPS belong to the euascomycete synthase (EAS) clade, which was shown to be expanded in euascomycete fungi and to contain primarily multimodular NRPSs, many of which may have niche‐specialized functions (Bushley & Turgeon, 2010) (Figure 4; purple box). The eleven adenylation (A) domain from *C. rosea* NPS4 clustered within the large clade containing peptaibol synthestases (Figure 4; peptaibol synthases clade), including TEX1 from *Trichoderma virens* (Figure 4; peptaibol synthases clade). Three of the A domains from *C. rosea* NPS5 grouped in a clade containing NPS8 from the corn pathogen *Cochliobolus heterostrophus* and peramine synthase from *Epichlӧe/Neotyphodium* endophytes of grasses (Figure 4; ChNPS8/peramine synthases). However, the remaining two A domains (2 and 3) of *C. rosea* NPS5 grouped closely with the second module of PS1 and PS4, two NRPSs involved in synthesis of ergot alkaloids in *Claviceps purpurea*.

### 3.6 Gene expression analyses of *nps4* and *nps5*

**FIGURE 5:**
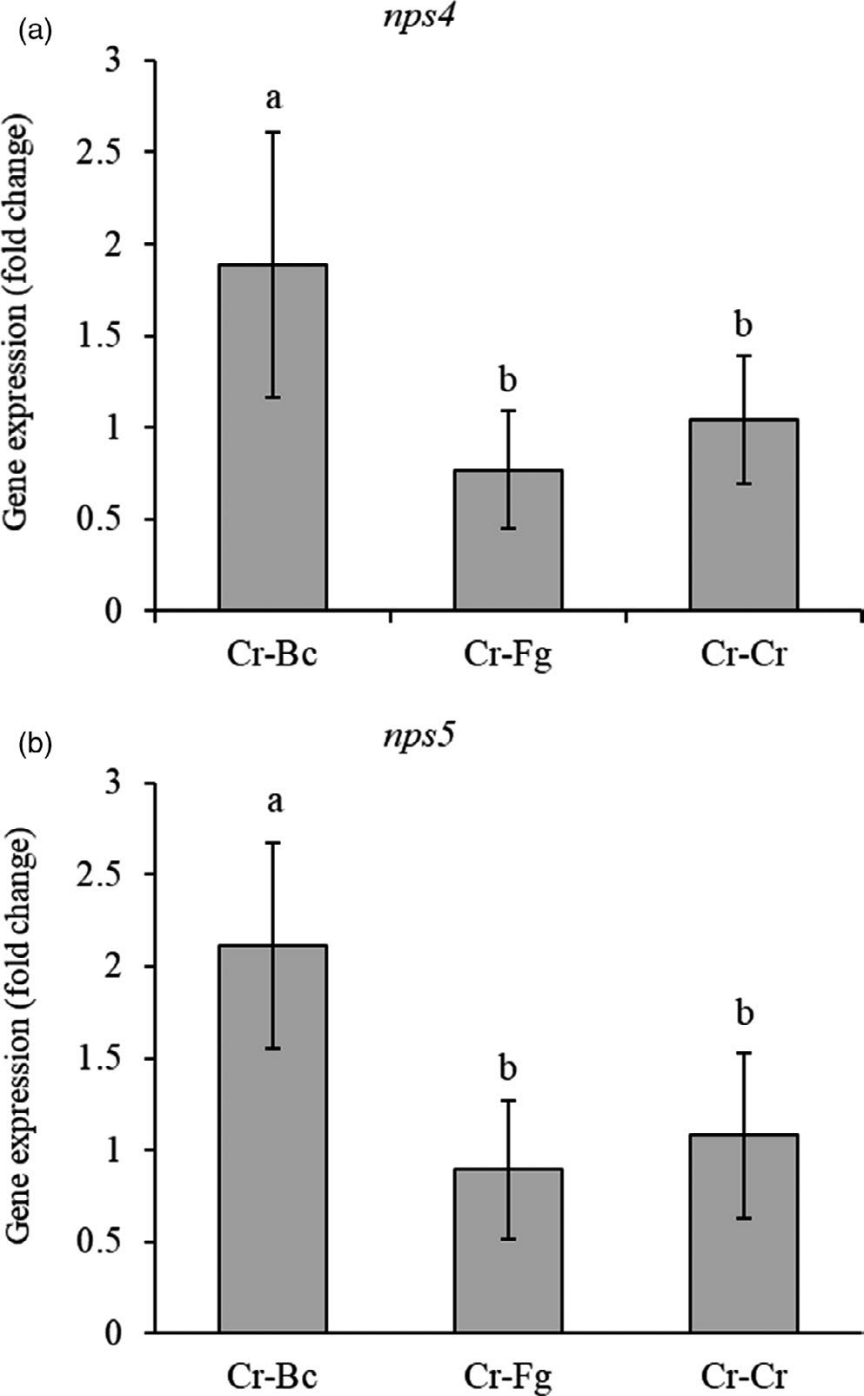
Expression analyses of *nps4* and *nps5* genes during interaction with fungal prey. Gene expression of (a) *nps4* and (b) *nps5* was estimated using RT‐qPCR during the confrontation of *Clonostachys rosea* with *Botrytis cinerea* (Cr–Bc), *C. rosea* with *Fusarium graminearum* (Cr–Fg) and *C. rosea* with itself (Cr–Cr, control). Relative expression was calculated as the ratio between the *nps4* and *nps5* genes and tubulin using the 2^–ΔΔCT^ method. Gene expression during the interaction of Cr–Bc was significantly higher (*p* ≤ .05) than that during the interaction of Cr–Cr or Cr–Fg, as determined by the Tukey–Kramer test. Error bars represent the standard deviation based on five biological replicates

The primer pair NPS4 F_val, NPS4 R_val and NPS5 F_val, NPS5 R_val (Table S1) specifically amplified a 149 bp and 179 bp amplicon from *nps4* and *nps5*, respectively. The relative expression for both *nps* genes was twofold higher during the confrontation between *C. rosea* and *B. cinerea* (*p* ≤ .01), but not with *F. graminearum*, compared with the self‐confronting control treatment (Figure 5a,b).

### 3.7 Generation and validation of *nps4* and *nps5* deletion strains

The *nps4* and *nps5* genes were replaced with the hygB selection cassette by homologous recombination using ATMT in *C. rosea* IK726. In total, 150 hygromycin‐resistant *C. rosea* colonies were obtained during transformation on selection plates containing cefotaxime and hygromycin. After repeated sub‐culturing of colonies on fresh selection plates, 130 hygromycin‐resistant colonies remained. Successful PCR amplification of products of the expected size using primers located upstream and downstream of the deletion construct in combination with primers located in the hygB cassette (Figures S1a and S2a) was observed for eight Δ*nps4* and Δ*nps5* strains, while no amplification was observed in the wild type (Figures S1b,c and S2b,c). RT‐PCR analysis further confirmed the loss of *nps4* and *nps5* expression in five randomly selected mutants (Δ*nps4*‐A, Δ*nps4*‐B, Δ*nps4*‐C, Δ*nps4*‐D, Δ*nps4*‐E and Δ*nps5*‐A, Δ*nps5*‐B, Δ*nps5*‐C, Δ*nps5*‐D, Δ*nps5*‐E) from each gene, respectively, while expression was observed in the wild type (Figures S1d and S2d). *Hph* expression was detected in all Δ*nps4* and Δ*nps5* strains, while no amplification was observed in the wild type (Figures S1e and S2e). Successful RT‐PCR amplification of *act* in all mutants and wild type showed that the cDNA template was intact (Figures S1, f and S2f).

### 3.8 Phenotypic analyses

**FIGURE 6:**
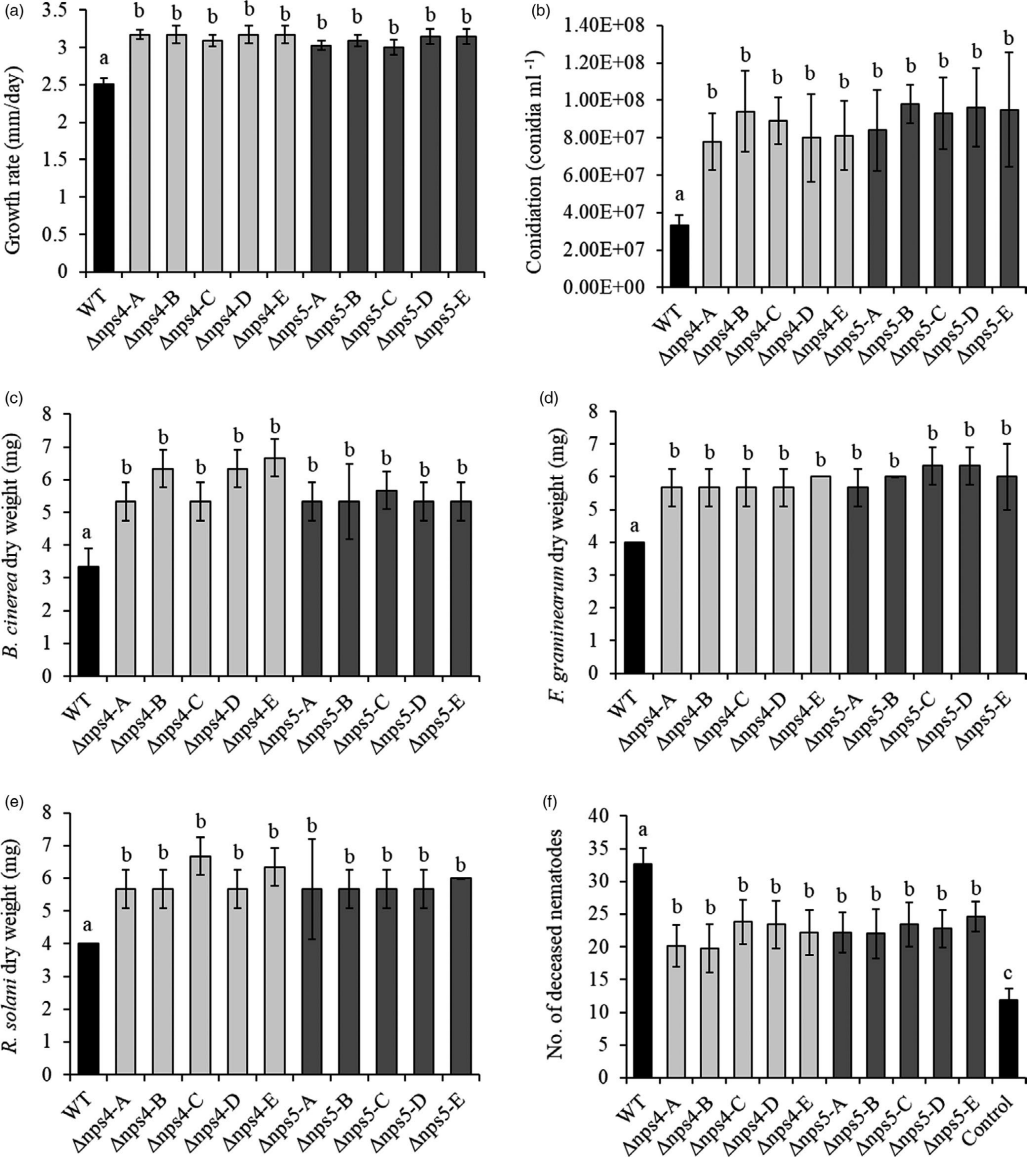
In vitro phenotypic analyses of *Clonostachys rosea* wild‐type, *nps4* and *nps5* deletion strains. (a, b) show growth and conidiation rates of *C. rosea* wild‐type, *nps4* and *nps5* deletion strains on potato dextrose agar (PDA). *C. rosea* wild‐type, *nps4* and *nps5* deletion strains were inoculated on PDA plates and incubated at 25°C. (a) Growth rate was measured daily up to seven days postinoculation (DPI). (b) Conidiation rate was calculated at 14 DPI. (c–f) show antagonistic tests in culture filtrates of *C. rosea* wild‐type, *nps4* and *nps5* deletion strains against *Botrytis cinerea* (c), *Fusarium graminearum* (d), *Rhizoctonia solani* (e) and the migratory endoparasitic nematode *Pratylenchus penetrans* in potato dextrose broth. An agar plug of *B. cinerea*, *F. graminearum* and *R. solani* was inoculated in culture filtrates produced by *C. rosea* wild‐type, *nps4* and *nps5* deletion strains and incubated at 25°C. Mycelia biomass production was determined by weighing the mycelial dry biomass at five DPI. Nematode mortality was assessed in culture filtrates from *C. rosea* wild‐type, Δ*nps4* and Δ*nps5* strains and in a control medium after 24 hr of incubation at 25°C. Error bars represent the standard deviation based on five biological replicates. Different letters indicate statistically significant differences (*p* ≤ .05) between treatments within an experiment based on the Tukey–Kramer test. Light and dark columns correspond to *nps4* and *nps5* deletion strains, respectively, while black column corresponds to control or wild‐type treatment

The growth rate of all *C. rosea nps4* and *nps5* deletion strains was on average 25% and 23% higher (*p* ≤ .001), respectively, than the wild‐type growth rate when grown on PDA plates (Figure 6a). Conidiation of *nps4* and *nps5* deletion strains was on average 156% and 182% higher (*p* ≤ .001), respectively, on PDA plates (Figure 6b).

Mycelial biomass of *B. cinerea*, *F. graminearum* and *R. solani* was significantly (*p* ≤ .019) increased (47%–71%) in culture filtrates from *C. rosea* Δ*nps4* and Δ*nps5* strains compared with filtrates from the wild‐type strain (Figure 6c‐e). On solid medium, growth rates of *B. cinerea*, *F. graminearum* and *R. solani* did not differ during interactions with *C. rosea* wild‐type, Δ*nps4* or Δ*nps5* strains, at either three or five DPI (Figure S3).

Nematicidal activity of culture filtrates from Δ*nps4* and Δ*nps5* strains was significantly weaker (*p* < .001) compared with the wild type; 24 hr of incubation in culture filtrates from *nps4* or *nps5* deletion strains resulted in 22% and 23% mortality, respectively, of *P. penetrans* nematodes compared with 33% mortality in wild‐type culture filtrates (Figure 6f). However, nematode mortality in the PDB control was even lower (12%, *p* ≤ .05) compared with either *C. rosea* strain (Figure 6f).

The metabolite profiles obtained by UHPLC‐MS were different for the wild‐type cultures compared to cultures of *nps4* and *nps5* deletion strains (Figure S4), as shown by the clustering obtained with PCA. However, a more detailed comparison between *nps4* or *nps5* deletion strains and wild‐type culture filtrates failed to identify any compounds that were produced exclusively in the wild‐type cultures. One unknown compound, with retention time (*t*_R_) 12 s and *m/z* 244.118 ([M + H]^+^), corresponding to the molecular formula (C_11_H_17_NO_5_), was produced at significantly higher levels in wild‐type cultures than in any of the mutant strains (wild type/*nps4*: 7.4, *p* < .001; wild type/*nps5* = 5.3, *p* < .001). The molecular formula and MSMS data do not fit any molecule in the database Dictionary of Natural Products.

### 3.9 Deletion of *nps4* and *nps5* reduces biocontrol efficacy against fusarium foot rot disease

**FIGURE 7:**
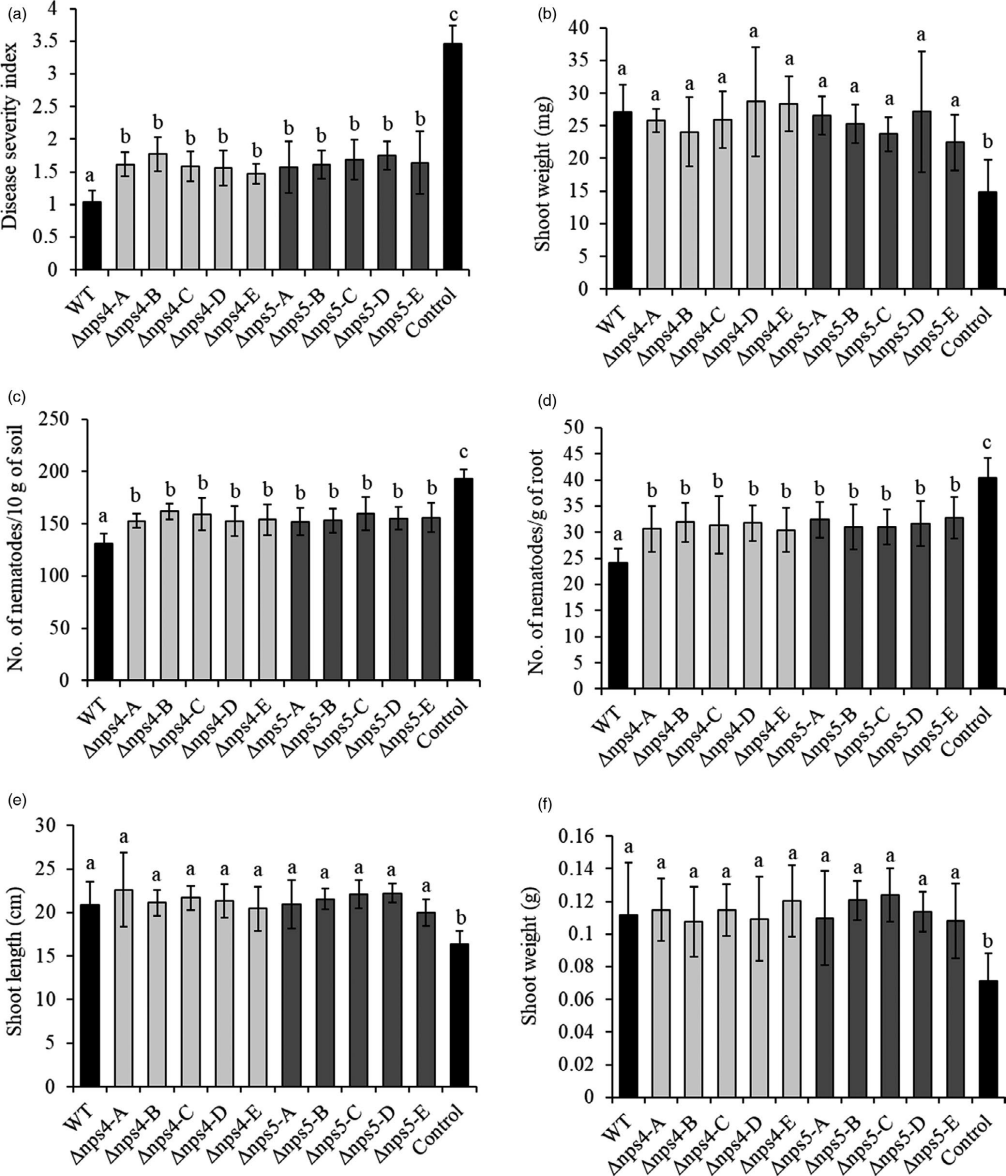
Effect of *Clonostachys rosea* wild‐type, *nps4* and *nps5* deletion strains on biocontrol of fusarium foot rot, nematode root disease and plant growth. (a) In vivo bioassay to test the biocontrol efficacy of *C. rosea* wild‐type, *nps4* and *nps5* deletion strains against *Fusarium graminearum* foot rot disease on wheat. Wheat seeds were coated with *C. rosea* conidia (wild‐type, Δ*nps4* or Δ*nps5* strains) and planted in moist sand together with an *F. graminearum* agar plug. Seedlings were harvested three weeks postinoculation, and disease symptoms were scored on a 0–4 scale. (b) Effect of *C. rosea* wild‐type, *nps4* and *nps5* deletion strains on dry shoot weight (g) of plants treated with *C. rosea* strains or plants that were not treated with any *C. rosea* strain (control). (c) Effect of soil applications of *C. rosea* wild‐type, Δ*nps4* and Δ*nps5* strains on the nematode population recovered from soil, including plant‐parasitic nematode genera (*Boleodorus*, *Helicotylenchus*, *Heterodera* (J2), *Merlinus*, *Paratylenchus*, *Pratylenchus*, *Rotylenchus* and *Tylenchorhynchus*) and other trophic groups (bacterivorous, fungivorous and omnivorous). (d) Effect of *C. rosea* wild‐type, *nps4* and *nps5* deletion strains on the nematode population recovered from wheat roots. Data include both migratory endoparasitic nematodes belong to genus *Pratylenchus* and endoparasitic nematodes from genus *Heterodera* (J2). The control treatment refers to plants grown in soil without *C. rosea* application. (e and f) show effect of soil applications of *C. rosea* wild‐type, *nps4* and *nps5* deletion strains on shoot length and shoot weight of wheat. (e) Average shoot length (cm) and (f) average dry shoot weight (g) of plants grown in soil treated with *C. rosea* wild‐type, *nps4* and *nps5* deletion strains. The control treatment refers to plants grown in soil without *C. rosea* application. For a and b, error bars represent the standard deviation based on five biological replicates and each replicate contained the mean of 12–15 plants while c–f error bars represent the standard deviation based on eight biological replicates. Different letters indicate statistically significant differences (*p* ≤ .05) between treatments within an experiment as determined by Fisher's least significant difference or Tukey–Kramer test. Light and dark columns correspond to *nps4* and *nps5* deletion strains, respectively, while black columns correspond to control or wild‐type treatment

Coating of wheat seeds with wild‐type *C. rosea* conidia provided adequate protection (*p* ≤ .05) against fusarium foot rot disease caused by *F. graminearum* (Figure 7a). Seed coating with conidia from *nps4* or *nps5* deletion strains also protected against foot rot (*p* ≤ .05), but to a significantly (*p* ≤ .05) lower degree compared with the wild type (Figure 7a). All *C. rosea* strains (wild type, Δ*nps4* or Δ*nps5*) increased (*p* ≤ .05) shoot weight of wheat with on average 75% compared with the fusarium foot rot control treatment (Figure 7b).

### 3.10 Deletion of *nps4* and *nps5* reduces biocontrol efficacy against nematode root disease

Seven weeks after inoculating the soil with *C. rosea*, the number of nematodes extracted was 32% (wild type), 19% (∆*nps4*) and 20% (∆*nps5*) lower, respectively, compared to nematode numbers extracted from the control treatment (*p* < .001; Figure 7c). Soil application of *nps4* and *nps5* deletion strains reduced the number of nematodes to a significantly (*p* < .001) lesser degree (20%) than that of the wild‐type treatment (Figure 7c).

Moreover, inoculation of *C. rosea* wild type also significantly (*p* < .001) reduced the number of nematodes (*Pratylenchus* and *Heterodera* J2) recovered from roots of wheat by 40% compared with the control treatment (Figure 7d). However, adding *nps4* and *nps5* deletion strains to soil reduced the number of extracted nematodes by only 23% and 20%, respectively, from wheat roots compared with the control treatment, and to a significantly (*p* < .001) lower degree (29%) than the wild‐type treatment (Figure 7d).

The inoculation of either *C. rosea* wild‐type or *nps4* and *nps5* deletion strains in soil significantly increased the shoot length (30%) and dry shoot weight (59%) compared to the control treatment, but there were no differences among the *C. rosea* treatments (Figure 7e, f). Furthermore, control plants displayed yellowing of leaves and chlorosis but this was not observed in plants grown in soil treated with either *C. rosea* wild‐type or *nps4* and *nps5* deletion strains.

## 4 DISCUSSION

In the present study, we explored the natural variation in nematode in vitro antagonism among 53 strains of *C. rosea* to identify potential factors that influence the outcome of biocontrol interactions in *C. rosea*. We found a significant variation between *C. rosea* strains in terms of in vitro antagonism against the root lesion nematode *P. penetrans*, suggesting quantitative and/or qualitative differences in their ability to produce secreted nematicidal compounds or enzymes. Meyer et al. (2004) also reported large variation in PDB culture filtrate toxicity towards *H. glycines* and *Meloidogyne incognita* nematodes between different strains of the same species, including *Cladosporium cladosporioides*, *F. equiseti* and *F. solani*. In addition, the level of in vitro antagonism against the migratory endoparasitic *P. penetrans* correlated well with various measures of antagonism against the sedentary endoparasitic *H. glycines*, indicating a broad‐spectrum activity of the responsible compounds or enzymes. A similar correlation (although weak) was reported for nematicidal activities against *H. glycines* and *M. incognita* (Meyer et al., 2004).

Previously, it was shown that *C. rosea* strain IA produced an epipolysulfanyldioxopiperazine compound with nematicidal activity against *Caenorhabditis elegans* and *Panagrellus redivivus* when grown on wheat kernels for 20 days (Dong et al., 2005). Culture filtrates from *C. rosea* strain IK726 grown in PDB exhibited nematicidal activity against a mixed community of plant‐parasitic nematodes (Iqbal, Dubey, McEwan, et al., 2018). Additionally, *C. rosea* strain 611 grown on PDB produced the extracellular serine protease PrC with nematicidal activity against *P. redivivus* (Li, Yang, Huang, & Zhang, 2006), showing that not only compounds, but also enzymes, are involved in *C. rosea* antagonism against nematodes.

An unexpected observation was that the culture filtrates from certain *C. rosea* strains resulted in lower *P. penetrans* mortality, but to some extent higher *H. glycines* egg hatching, compared with the PDB control treatment. A similar stimulatory effect on *H. glycines* and *M. incognita* egg hatching was reported previously from a range of different fungi (Meyer et al., 2004) and explained by either production of specific compounds stimulating egg hatch or fungal utilization of PDB compounds inhibiting egg hatch (Chen, Dickson, & Mitchell, 2000; Meyer et al., 2004). The previously mentioned *prC* protease gene in *C. rosea* is induced by the pH‐responsive PacC transcription factor, and *pacC* itself is repressed by low (3–4) pH (Zou, Tu, et al., 2010). As *C. rosea* can lower the pH of the growth medium (Fatema et al., 2018), we speculate that low nematode mortality in certain *C. rosea* culture filtrates may be connected with variation in the pH response, although this hypothesis needs to be further investigated. However, the pH itself is reported not to influence egg hatching of *H. glycines* (Pike et al., 2002) or a direct cause of antagonism against *M. incognita* (Djian, Pijarowski, Ponchet, Arpin, & Favre‐Bonvin, 1991).

Interestingly, egg parasitism also correlated with production of nematicidal compounds or enzymes, suggesting that antibiosis and/or enzymatic digestion of the egg shell may contribute to direct parasitism. The result from the current study further confirms the ability of *C. rosea* to directly parasitize nematodes reported previously by Zhang et al. (2008). They showed that pathogenesis starts from attachment of conidia to the nematode cuticle, followed by penetration into the nematode leading to death and degradation of the nematode.

Although high in vitro antagonism was observed in our study, the correlation with biocontrol activity in soil was weak. Although *C. rosea* strains with high in vitro antagonism against *P. penetrans* performed better (at *p* = .052) in reducing plant‐parasitic nematode numbers in soil compared with strains with low in vitro antagonism, no significant differences between the groups showing low and high in vitro antagonism were observed in terms of the nematode numbers in roots or in plant biomass. This is not surprising given the complexity of both biological control mechanisms, including not only antibiosis, but also competition for nutrients and space, direct parasitism and induction of plant defence reactions (Harman, Howell, Viterbo, Chet, & Lorito, 2004; Jensen, Karlsson, & Lindahl, 2017), and natural soil environments. However, most *C. rosea* strains were able to control the populations of plant‐parasitic nematodes in the potted plant assay. This decline in nematode populations positively correlated with improved growth of wheat plants and lack of disease symptoms, such as chlorosis and yellowing of leaves, which is usually associated with root damage by nematodes (Jones et al., 2013). These results are in agreement with previous findings that showed that inoculation of *C. rosea* strain IK726 into soil reduced the number of plant‐parasitic nematodes by 40% – 70% and resulted in better growth of carrot and wheat (Iqbal, Dubey, McEwan, et al., 2018). Other biocontrol fungi, such as *T. koningii* and *T*. confer (cf.) *harzianum,* are also reported to control the root‐knot nematode *M. arenaria* and the citrus nematode *Tylenchulus semipenetrans,* respectively (Reddy, Rao, & Nagesh, 1996; Sharon et al., 2001; Windham, Windham, Williams, & Wallace, 1989). SCN egg‐parasitic fungi such as *C. rosea* and *Pochonia chlamydosporia* (syn. *Metacordyceps chlamydosporia*) were isolated from females and cysts (Hu, Samac, Liu, & Chen, 2017; Hu, Strom, Haarith, Chen, & Bushley, 2018; Toju & Tanaka, 2019) and shown to have the potential to control and regulate the nematode populations (Hamid et al., 2017; Hu et al., 2017). A few *C. rosea* strains failed to control plant‐parasitic nematodes in the current assay. A certain level of strain‐to‐strain variation in biocontrol ability has been previously reported in *Trichoderma* and *Clonostachys* (Hermosa et al., 2000; Knudsen et al., 1997).

The significant and continuous variation in nematicidal activity of *C. rosea* culture filtrates against *P. penetrans* suggested polygenic inheritance of nematode antagonism and that a GWA of the trait is possible. Using an empirical Bayes large‐scale hypothesis testing approach (Stephens, 2016), we were able to identify 279 SNP markers that were significantly associated with in vitro nematode antagonism in *C. rosea*. Production of, and tolerance to, secondary metabolites is one important feature of antagonism and biocontrol in *C*. *rosea* (Demissie, Foote, Tan, & Loewen, 2018; Dubey et al., 2014, 2016; Fatema et al., 2018; Iqbal et al., 2019; Karlsson et al., 2015; Nygren et al., 2018). Therefore, genes predicted to encode proteins involved in secondary metabolite biosynthesis and transport that were physically located close to SNPs segregating with nematicidal activity were considered as candidate biocontrol factors.

The three identified putative MFS transporters (CRV2G00021063, CRV2G00021188 and CRV2G21796) were classified in MFS transporter families 2.A.1.2.33, 2.A.1.14.11 and 2.A.1.2.85, respectively. MFS transporters in family 2.A.1.2.33 have unknown function, and no member of this family has been characterized in detail. Members of the 2.A.1.14.11 family have been shown to be involved in nicotinic acid import across the plasma membrane in *Saccharomyces cerevisiae* (Llorente & Dujon, 2000), while a member of MFS transporter family 2.A.1.2.85 was shown to be localized in the peroxisomal membrane and is required for transport of isopenicillin N, a precursor of penicillin antibiotic biosynthesis, in the fungus *Penicillium chrysogenum* (Fernández‐Aguado, Ullán, Teijeira, Rodríguez‐Castro, & Martín, 2013). Interestingly, all three families evolve under selection for increased paralog gene numbers in *C. rosea* (Nygren et al., 2018), suggesting that these MFS transporters contribute to ecological niche adaptation (Wapinski, Pfeffer, Friedman, & Regev, 2007). In addition, one SNP was identified in the ABC transporter gene *abcb14* (CRV2G00020959) predicted to be involved in multidrug resistance (Karlsson et al., 2015).

Several SNPs were associated with genes predicted to be involved in biosynthesis of secondary metabolites in other fungal species; CRV2G00013596 was predicted to be a fungal‐type zinc cluster transcription factor with sequence similarity towards the *Alternaria cinerariae* DHC4 regulator of dehydrocurvularin biosynthesis (Cochrane et al., 2015), CRV2G00022270 showed sequence similarity with a decarboxylase involved in biosynthesis of yanuthone D in *Aspergillus niger* that acts as an antibiotic against fungi and bacteria (Holm et al., 2014), and CRV2G00019702 had sequence similarity towards the FTMP450‐2 fumitremorgin C synthetase involved in alkaloid biosynthesis in *A. fumigatus* (Kato et al., 2009; Maiya, Grundmann, Li, & Turner, 2006).

Involvement of NRPSs in the biosynthesis of nematicidal compounds has been suggested previously in *C. rosea* (Dong et al., 2005; Iqbal et al., 2019; Karlsson et al., 2015). Therefore, the identified *nps4* and *nps5* genes were studied in more detail. The predicted modular structures of NPS4 and NPS5 contained all the essential domains, that is A, T and C, which are necessary for a functional NRPSs to carry out peptide synthesis (Conti, Stachelhaus, Marahiel, & Brick, 1997; Keating, Marshall, & Walsh, 2000; Keating et al., 2002; May et al., 2002; Stachelhaus, Mootz, & Marahiel, 1999; Weber, Baumgartner, Renner, Marahiel, & Holak, 2000). The domain structure and phylogenetic placement of NPS4 strongly suggests that it is a peptaibol synthase of eleven A‐T‐C modules. Peptaibols have previously been shown to be produced by various fungi, including *C. rosea* (Rodríguez et al., 2011), species of Trichoderma (Degenkolb et al., 2012; Wiest et al., 2002), as well as the potential nematode parasite *Purpureocillium lilacinum* (Wang et al., 2016), the insect parasite *Tolypocladium geodes* (Tsantrizos, Pischos, & Sauriol, 1996) and the related truffle parasite *T. ophioglossoides* (Quandt, Di, Elser, Jaiswal, & Spatafora, 2016). They have canonically been considered to have roles in mycoparasitism or antifungal activity (Schirmböck et al., 1994), and some studies have demonstrated activity against the oomycete pathogen *Phytopthora* spp. (Sansom, Balaram, & Karle, 1993). Peptaibols are amphipathic compounds, and their antimicrobial function results from formation of ion channels in cell membranes (Sansom et al., 1993). In this study, the increased expression of *nps4* in confrontation with the fungal pathogen *B. cinerea* and reduced disease severity in pot assays with the fungal root rot pathogen *F. graminearum* confirm a potential role for peptaibols in antagonism and biocontrol of fungal pathogens. While peptaibols have not previously been shown to be involved in activity against nematodes, the presence of large peptaibol synthases in *C. rosea* IK726 and the nematode egg‐parasitic fungus *P. lilacinum,* coupled with the decreased antagonism towards nematodes observed in vitro in this study, suggests they should be further investigated for activity against plant‐parasitic nematodes.

Phylogenetic analysis also placed NPS5 within the euascomycete clade, and three A domains grouped with NPS8 from the corn pathogen *C. heterostrophus* and peramine synthase. Peramine synthase was first identified in symbiotic Epichlӧe/Neotyphodium endophytes of grasses and has been shown to produce a pyrrolopyrazine compound that has activity against herbivorous insects (Tanaka, Tapper, Popay, Parker, & Scott, 2005). The other two A domains of NPS5 group adjacent to a clade containing the second A domain of two NRPSs (PS1 and PS4) that are involved in the biosynthesis of ergot alkaloids. Like peramine, ergot alkaloids have bioactivity against insects and animals (Cross, 2003), and both their evolutionary history (Spatafora, Sung, Sung, Hywel‐Jones, & White, 2007; Torres, Singh, Vorsa, & White, 2008) and experimental evidence (Panaccione et al., 2006; Potter, Tyler Stokes, Redmond, Schardl, & Panaccione, 2008) suggest they may also be involved in protection against herbivory. However, while the presence of *Neotyphodium* endophytic fungi has been shown to reduce populations of the root lesion nematode, *P. scribneri*, this effect could not be attributed to ergot alkaloids as genetic knockout strains of ergot alkaloid NRPSs of the endophyte did not lower nematode colonization *in planta* and only the ergot alkaloid compound ergovaline had any inhibitory effect on nematode mobility (Panaccione et al., 2006). While it is challenging to predict the chemical class of compounds produced by NPS5, it is interesting that it shares an evolutionary relationship with NRPSs producing compounds with activity against other invertebrate pests such as insects.

Higher expression of both *nps4* and *nps5* during a confrontation with *B. cinerea*, but not against *F. graminearum*, suggests a role of both NRPSs during mycoparasitism of *B. cinerea*. The *C. rosea* NRPS genes *nps1* and *nps13* are also induced specifically during interaction with *B. cinerea*, but not with *F. graminearum* (Iqbal et al., 2019; Nygren et al., 2018), suggesting that only interaction with specific antagonistic fungi may trigger expression. NRPS gene expression is also reported to be induced in the truffle‐parasitizing fungus *T. ophioglossoides* where the NRPS‐like gene TOPH 03459 was highly upregulated in response to secreted material from the truffle host *Elaphomyces muricatus* (Quandt et al., 2016).

Our result indicates that the metabolites produced by NPS4 and NPS5 may be toxic for the fungus itself and exhibit an inhibitory effect on *C. rosea*, as deletion of *nps4* and *nps5* resulted in increased growth and conidiation. Alternatively, deletion of *nps4* and *nps5* may lead to redistribution of resources, from secondary metabolite biosynthesis to growth and conidiation (Calvo, Wilson, Bok, & Keller, 2002), that is a trade‐off between allocating resources to growth versus secondary metabolite production. Our results are in agreement with previous finding that deletion of the *nps1* NPRS gene in *C. rosea* strain also increased growth rate and conidiation (Iqbal et al., 2019). Similar results were also reported in the mycoparasitic fungus *T. virens* where deletion strains of NRPS genes, that is *tex7*, *tex8, tex10* and *tex13,* resulted in increased growth rate compared with the wild type (Mukherjee, Buensanteai, Moran‐Diez, Druzhinina, and Kenerley (2012); Mukherjee et al., 2018;. Our results are also in line with previous findings where deletion of the polyketide synthetase genes *pks22* and *pks29* in *C. rosea* resulted in increased conidiation, indicating that secondary metabolites may regulate growth and development in *C. rosea* (Fatema et al., 2018).

The significant increase in *B. cinerea*, *F. graminearum* and *R. solani* mycelial biomass and reduced nematode mortality when grown in culture filtrates of Δ*nps4* and Δ*nps5* strains compared with the wild type suggest that NPS4 and NPS5 biosynthesize metabolites with broad‐spectrum antagonistic properties against these organisms. Previously, nine verticillin‐type epipolysulfanyldioxopiperazine (gliocladin A–E, verticillin A, 11‐deoxyverticillin A, Sch52900 and Sch52901) compounds were isolated from *C. rosea* strain 1A and all compounds showed toxicity against *C. elegans* and *P. redivivus* nematodes and more than 50% mortality rate was observed following 24 hr of incubation (Dong et al., 2005). Disruption of the hybrid PKS/NRPS zeamine (*zmn*) from the gram‐negative bacterium *Serratia plymuthica* strain A153 resulted in attenuation of the nematicidal activity against *C. elegans* (Hellberg, Matilla, & Salmond, 2015). Culture filtrates from the Δ*nps4* and Δ*nps5* strains still exhibited a certain degree of nematicidal activity that might be due to the production of auxiliary compounds or enzymes, such as serine proteases, contributing to the nematicidal activity. As mentioned before, *C. rosea* strain 611 produced the extracellular serine protease PrC during growth on PDB that caused 80% mortality of *P. redivivus* during incubation for 48 hr (Li et al., 2006).

NPS4 and NPS5 are predicted to contain eleven and five NRPS modules, respectively, suggesting the corresponding nonribosomal peptides to be constructed from eleven and five building blocks, respectively. When comparing wild‐type cultures with *nps4* and *nps5* deletion strains, the corresponding peptides should be absent in the mutant strains, but present in wild‐type cultures. However, no compound was found to be exclusively produced in wild type and not present in the *nsp4* or *nsp5* deletion strains. The compound with molecular formula C_11_H_17_NO_5_ at *t*_R_ 12 s was found at significantly higher concentrations in wild‐type cultures compared to mutant cultures, but the formula does not support a peptide type of structure, and it was present in all mutant strains. There are several possible explanations to why the expected peptides were not observed in the wild‐type cultures, for example that *nsp4* and *nps5* may not be highly expressed during normal laboratory conditions but only during biotic or abiotic stress. This is supported by gene expression data, which showed *nsp4* and *nsp5* to be upregulated during interaction with *B. cinerea*. The peptides may also be produced at very low levels and thus escape detection, and this could be the case if only very low concentrations of the putative peptides are needed to obtain the desired effects on an interacting organism such as *B. cinerea* or nematodes.

NPS4 and NPS5 are required for full biocontrol ability of *C. rosea* to control foot rot disease caused by *F*. *graminearum* and nematode root disease in wheat. Together with previous result where deletion of the *nps1* NRPS gene in *C. rosea* attenuated the antagonistic and biocontrol potential of *C. rosea* (Iqbal et al., 2019), this emphasizes the importance of NRPSs for the biocontrol ability of *C. rosea*. However, soil inoculation of the *nps4* and *nps5* deletion strains still resulted in increased shoot length and shoot weight of wheat plants in accordance with previous work (Iqbal, Dubey, McEwan, et al., 2018), possibly indicating that the plant growth‐promoting effect in *C. rosea* (Ravnskov et al., 2006; Roberti et al., 2008) is mechanistically separated from biocontrol of pathogens.

In the present study, we demonstrated that intrinsic differences exist among *C. rosea* strains in terms of in vitro antagonism against *P. penetrans* nematodes. We further showed that these strain differences were also reflected in antagonism against *H. glycines*, suggesting a broad‐spectrum mechanism. We also showed that GWA can be used to identify candidate genes for biocontrol‐related phenotypes, such as in vitro antagonism, reduced disease symptoms and reduced pathogen populations. Finally, we showed that the *nps4* and *nps5* gene products partially contribute to in vitro antagonism and biocontrol in *C. rosea*.

## CONFLICT OF INTEREST

None declared.

## Supporting information

Fig S1

Fig S2

Fig S3

Fig S4

Table S1

Table S2

## Data Availability

All data generated or analysed during this study are included in this manuscript and its supporting files.
