# Supplementary figures and images for: Natural variation of root lesion nematode antagonism in the biocontrol fungus Clonostachys rosea and identification of biocontrol factors through genome‐wide association mapping
Source: Evol Appl. 2020 Jun 2;13(9):2264–83. doi: 10.1111/eva.13001 (PMC7513725; doi:10.1111/eva.13001)

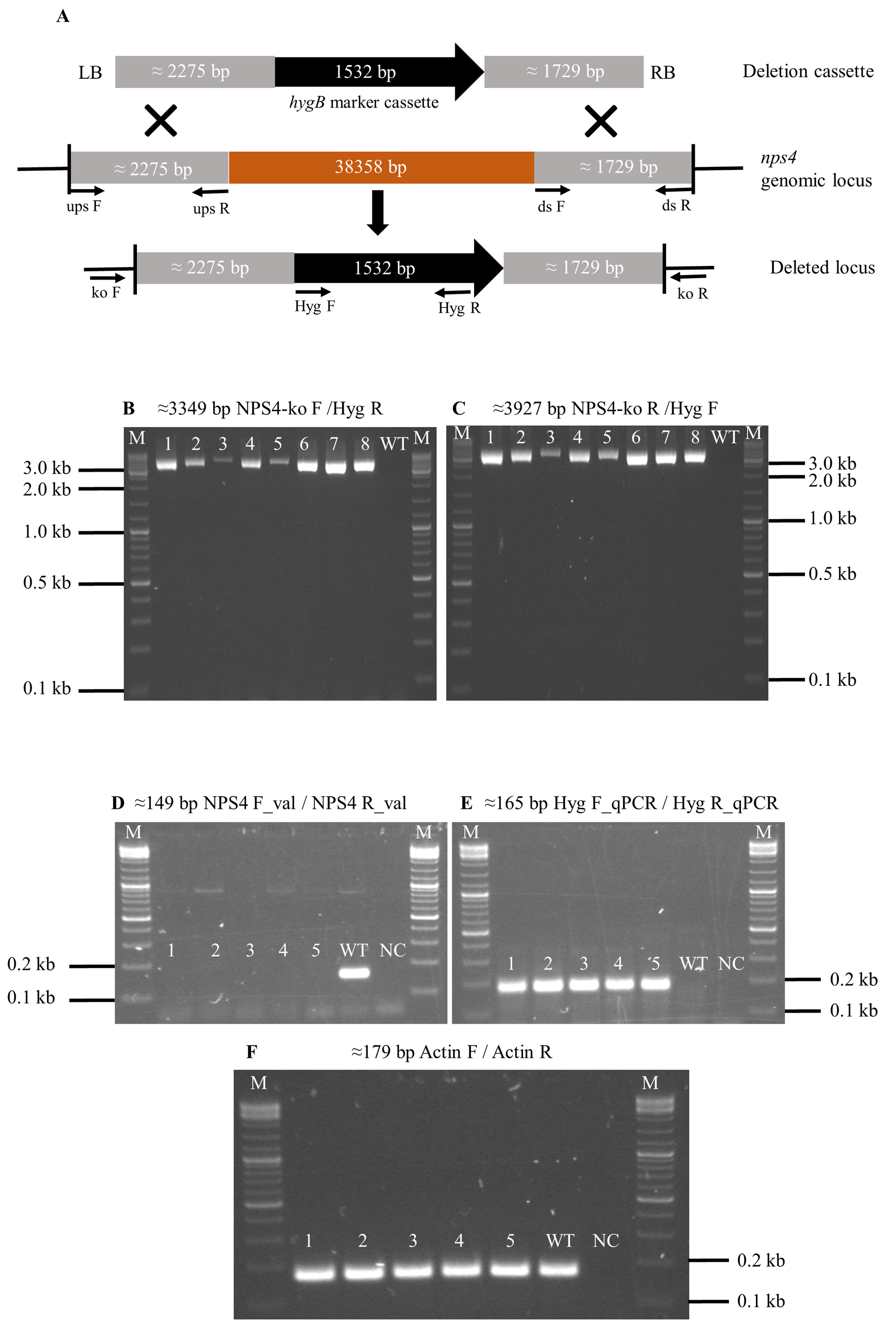

Supplement: Supplementary file 1 — Fig S1 [file EVA-13-2264-s001.tif]

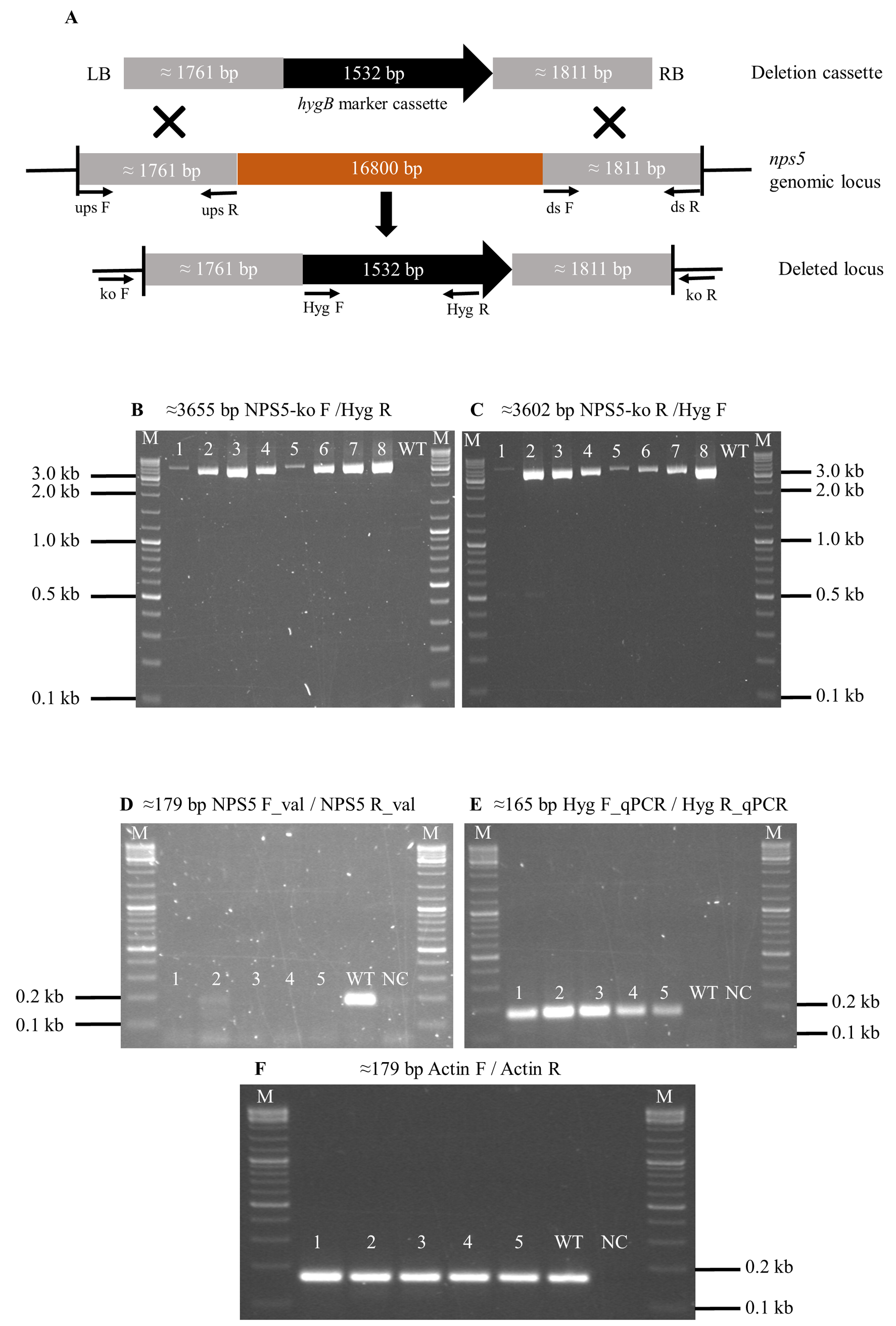

Supplement: Supplementary file 2 — Fig S2 [file EVA-13-2264-s002.tif]

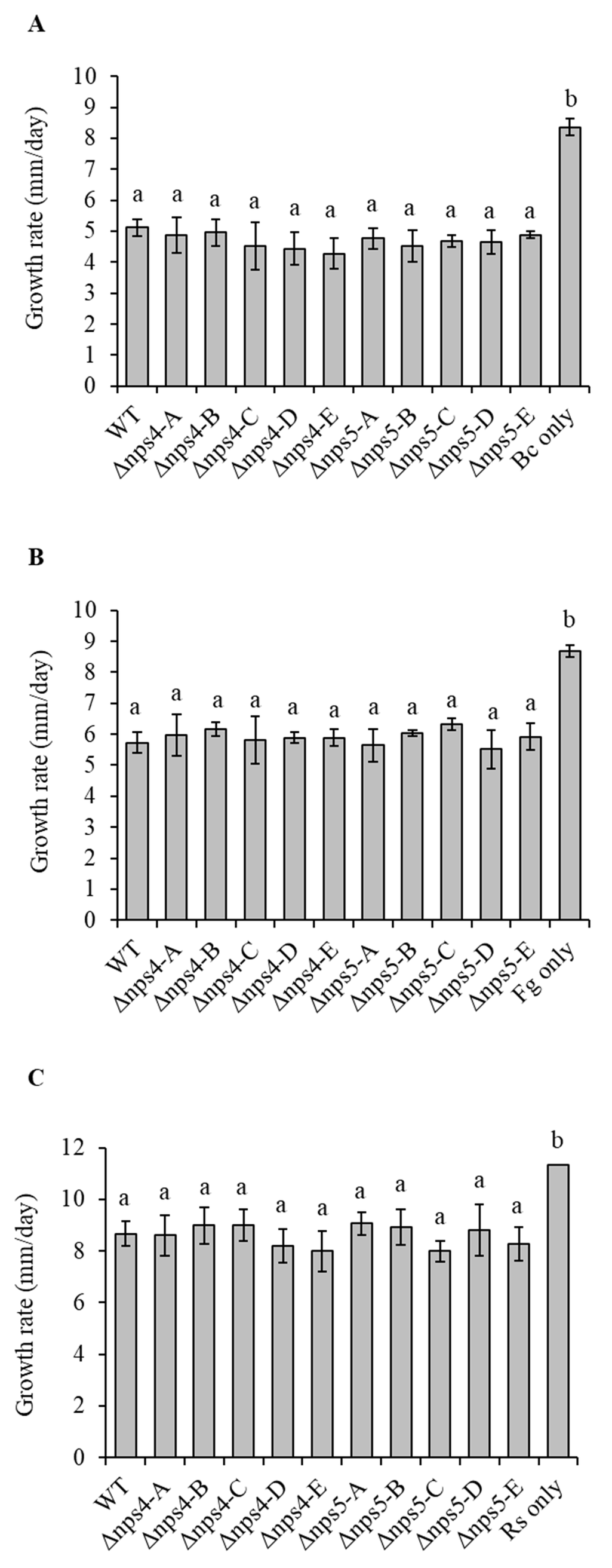

Supplement: Supplementary file 3 — Fig S3 [file EVA-13-2264-s003.tif]

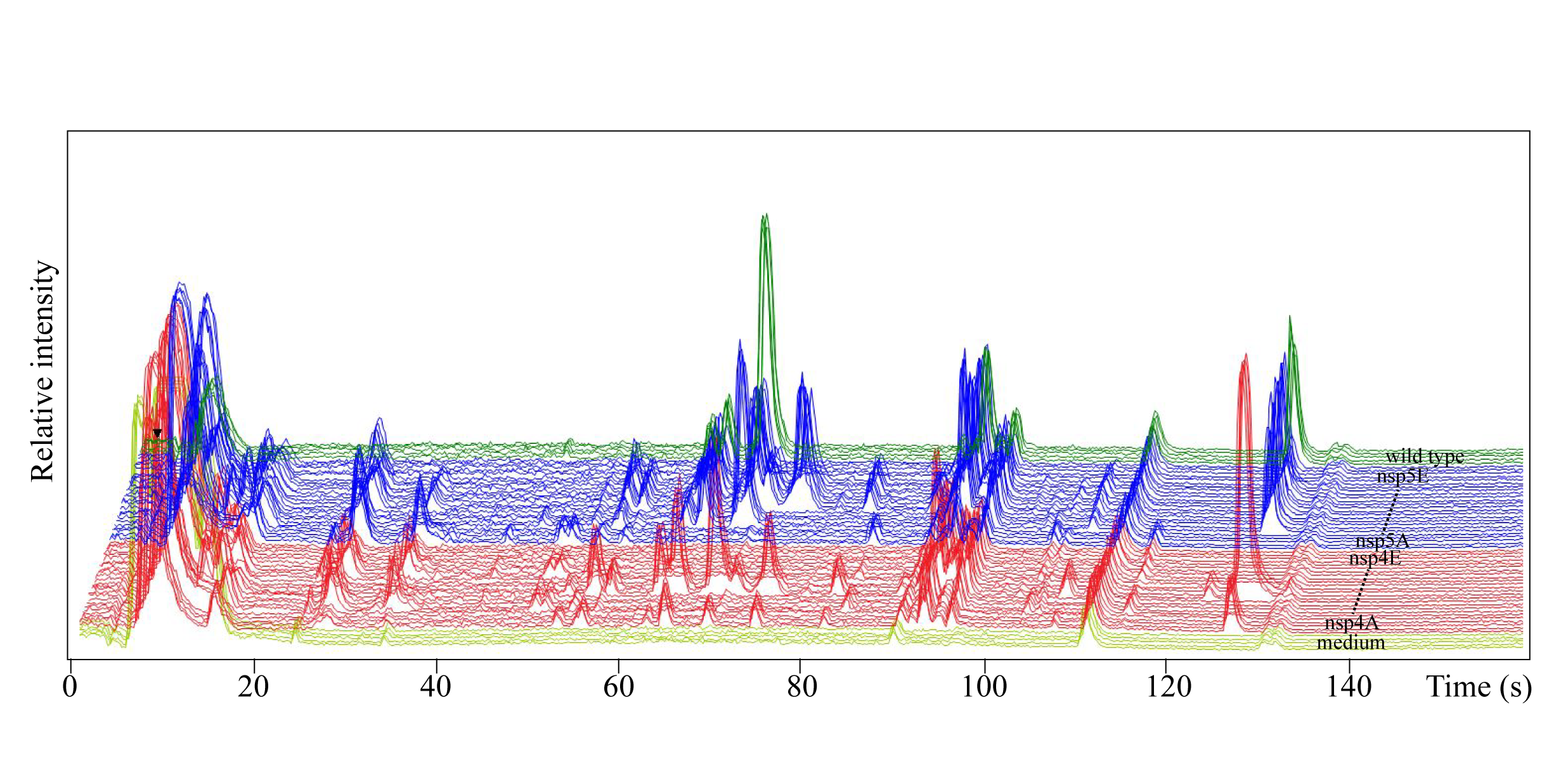

Supplement: Supplementary file 4 — Fig S4 [file EVA-13-2264-s004.tif]
