# Supplementary material for: Natural variation of root lesion nematode antagonism in the biocontrol fungus Clonostachys rosea and identification of biocontrol factors through genome‐wide association mapping
Source: Evol Appl. 2020 Jun 2;13(9):2264–83. doi: 10.1111/eva.13001 (PMC7513725; doi:10.1111/eva.13001)
Supplement: Supplementary file 5 — Table S1 [file EVA-13-2264-s005.docx]

**TABLE S1.** **List of primers used in this study.**

| **Name** | **Target gene** | **Sequences (5′ → 3′)** |
| --- | --- | --- |
| Tub CF | *tub* | ^a^ttc aga ccg gtc agt gcg ta |
| Tub CR |  | ^a^gcc aga aag cag ca caa t |
| ActinF_qPCR | *actin* | gtt ctg gat tcc ggt gat ggt gtc |
| ActinR_qPCR |  | tcg gca gtg gtg gag aag gtg t |
| NPS4-ups F | *nps4* | ^b^ggg gacaagtttgtacaaaaaagcaggcttaagccgtaaggactctgatgactcg |
| NPS4-ups R |  | ^b^ggggacaactttgtatagaaaagttgggtgagtcgtgctcgctgggctatt |
| NPS4-ds F | *nps4* | ^b^ggggacaactttgtataataaagttgtaacaggatggtggcgagacaatg |
| NPS4-ds R |  | ^b^ggggaccactttgtacaagaaagctgggttgaaaatcctgcccaaaaccacc |
| NPS4-ko F | *nps4* | tca gtc agc cgc tct atc cct aca |
| NPS4-ko R |  | tcg cgg aga agt tca gca gat t |
| NPS5-ups F | *nps5* | ^b^ggggacaagtttgtacaaaaaagcaggcttattggcaaagagctcgatgttcac |
| NPS5-ups R |  | ^b^ggggacaactttgtatagaaaagttgggtgggcttggcacgggtcagga |
| NPS5-ds F | *nps5* | ^b^ggggacaactttgtataataaagttgtaacgacaacaatgcgcctgataaag |
| NPS5-ds R |  | ^b^ggggaccactttgtacaagaaagctgggtttgcctggcctcctcaataaacat |
| NPS5-ko F | *nps5* | tgc ttc atc cat cag cct acc c |
| NPS5-ko R |  | gct ccc aga agc gaa cca cac |
| Hyg F | *hph* | ^a^gcg cgc aat taa ccc tca c |
| Hyg R |  | ^a^gaa ttg cgc gta cag aac tcc |
| HygF_qPCR | *hph* | ^a^acg gcg gga gat gca ata ggt |
| HygR_qPCR |  | ^a^gct tcg atg tag gag ggc gtg g |
| NPS4 F_val | *nps4* | ^c^atg ttg ggt ggc tgt gta tgt att cc |
| NPS4 R_val |  | ^c^cat ttt gag gct cgg gta atc ttc c |
| NPS5 F_val | *nps5* | ^c^cat ccg ggg ata ttt gtc ttg tgt t |
| NPS5 R_val |  | ^c^gtc ggg att ctt ctt tgc tta tgc t |

^a^*tub* primers and *hph* primers were retrieved from Mamarabadi et al. (2008) and Dubey et al. (2016), respectively.

^b^attB and attBr sequences are underlined.

^c^The same primers were used for gene expression analyses.

Abbreviation used for genes: *tub* *=* β-tubulin; *hph* = hygromycin B phosphotransferase; *nps* = non-ribosomal peptide synthetase.
